# Quantum chemical accuracy from density functional approximations via machine learning

**DOI:** 10.1038/s41467-020-19093-1

**Published:** 2020-10-16

**Authors:** Mihail Bogojeski, Leslie Vogt-Maranto, Mark E. Tuckerman, Klaus-Robert Müller, Kieron Burke

**Affiliations:** 1 Machine Learning Group, Technische Universität Berlin, Marchstr. 23, 10587 Berlin, Germany; 2 Department of Chemistry, New York University, New York, NY 10003 USA; 3 Courant Institute of Mathematical Science, New York University, New York, NY 10012 USA; 4 NYU-ECNU Center for Computational Chemistry at NYU Shanghai, 3663 Zhongshan Road North, Shanghai, 200062 China; 5 Department of Artificial Intelligence, Korea University, Anam-dong, Seongbuk-gu, Seoul, 02841 Korea; 6 Max-Planck-Institut für Informatik, Stuhlsatzenhausweg, 66123 Saarbrücken, Germany; 7 Department of Physics and Astronomy, University of California, Irvine, CA 92697 USA; 8 Department of Chemistry, University of California, Irvine, CA 92697 USA

**Keywords:** Computational chemistry, Computational science

## Abstract

Kohn-Sham density functional theory (DFT) is a standard tool in most branches of chemistry, but accuracies for many molecules are limited to 2-3 kcal ⋅ mol^−1^ with presently-available functionals. Ab initio methods, such as coupled-cluster, routinely produce much higher accuracy, but computational costs limit their application to small molecules. In this paper, we leverage machine learning to calculate coupled-cluster energies from DFT densities, reaching quantum chemical accuracy (errors below 1 kcal ⋅ mol^−1^) on test data. Moreover, density-based *Δ*-learning (learning only the correction to a standard DFT calculation, termed *Δ*-DFT ) significantly reduces the amount of training data required, particularly when molecular symmetries are included. The robustness of *Δ*-DFT  is highlighted by correcting “on the fly” DFT-based molecular dynamics (MD) simulations of resorcinol (C_6_H_4_(OH)_2_) to obtain MD trajectories with coupled-cluster accuracy. We conclude, therefore, that *Δ*-DFT  facilitates running gas-phase MD simulations with quantum chemical accuracy, even for strained geometries and conformer changes where standard DFT fails.

## Abstract (web summary)

High-level ab initio quantum chemical methods carry a high computational burden, thus limiting their applicability. Here, the authors employ machine learning to generate coupled-cluster energies and forces at chemical accuracy for geometry optimization and molecular dynamics from DFT densities.

## Introduction

The recent rise in the popularity of machine-learning (ML) methods has engendered many advances in the molecular sciences. These include the prediction of properties of atomistic systems across chemical space^1–26^, the construction of accurate force fields^27–39^ for ML-based molecular dynamics (MD) simulations, the representation of the (high-dimensional) statistical distribution of molecular conformers^40–42^, or the prediction of the kinetics of structural transformation of materials^43^. In many applications, a key task for an ML model is to predict the outcome of an electronic structure calculation without the calculation’s having to be explicitly performed. This could be done at any desired level of electronic structure theory from density functional theory (DFT) to the current gold-standard, namely, coupled-cluster with single, double, and perturbative triple excitations (CCSD(T)). While the latter is generally preferable, its putative *N*^7^ computational scaling with system size makes it prohibitive for large molecular systems or even for small systems if many energy and energy gradient calculations are needed, as would be the case in MD simulations or geometry optimizations. Therefore, Kohn-Sham (KS) DFT, with its putative *N*^3^ scaling, is often employed as an acceptable compromise between computational efficiency and accuracy. Unfortunately, the wavefunction and DFT formalisms are so distinct that there is no known way to combine the accuracy of the former with the speed of the latter. Thus, an important advance could be achieved if the power of ML could be leveraged to allow large numbers of CCSD(T) calculations to be performed at a cost equal to or even less than that of the same number of DFT calculations for a given system.

An ML scheme capable of realizing the aforementioned objective should satisfy several important criteria: First, the ML framework should be able to deliver basic molecular properties, such as total energies, geometries, and, in principle, electronic properties, all at CCSD(T) accuracy. Beyond this, however, it should also allow geometry optimization and long time-scale MD to be performed with energies and forces at the CCSD(T) accuracy level. The construction of such an ML approach requires a molecular descriptor flexible enough to accomplish both types of tasks, and for this, it seems natural to employ the electron density. It is worth noting that as molecular descriptors have evolved from objects such as SMILES strings^44,45^, molecular graphs^46,47^, and molecular graphs with feature vectors^24,25,48^, there has been a progression toward descriptors that attempt to capture key features of the electron density in a simple manner^15,48–51^. Admittedly, employing the full electron density carries with it a considerable computational cost; nevertheless, it is useful to develop such frameworks, considering that more optimal algorithms could follow. Previously, we had shown that the electron density could be used in a self-consistent manner to train a system-specific density functional (akin to a system-specific force field^52^) using a mapping from the external potential to the electron density and a second map of the density to the total energy^53^. Rather than delivering a solution to the KS equations, the first map (denoted the ML-HK map) bypasses the KS equations in a manner that is akin to solving the original Hohenberg-Kohn functional differential equation^54^. The second map from density to energy predicts the result of plugging that solution back into the Hohenberg-Kohn functional to obtain the ground-state energy. While other machine-learning methods for the prediction of electron densities or density functionals have appeared recently^50,51,55–62^, the ML-HK map facilitates the use of both machine-learned densities, from which electronic properties could be computed, and density functionals for obtaining total energies and gradients for geometry optimization and MD simulation.

**Fig. 1:**
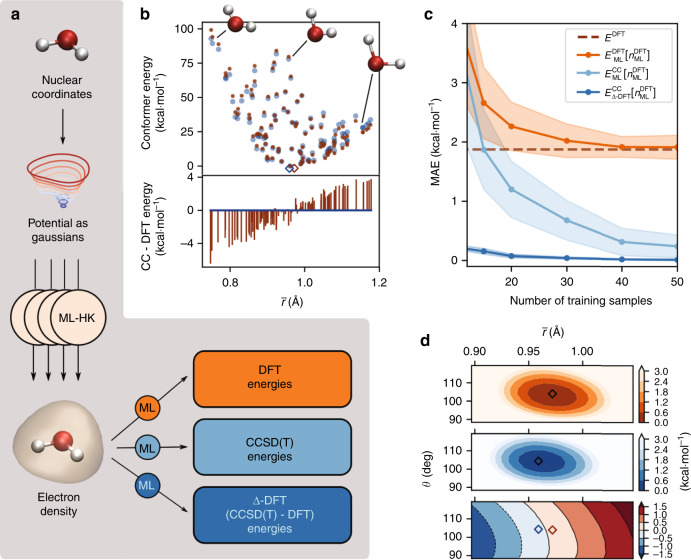
Illustration of density-based machine learning for water conformer energies. For all panels, DFT energies (orange) are shown alongside CC energies (blue) for the same molecular conformers, with optimized geometries indicated by open diamonds. **a** The nuclear potential, represented by an approximate Gaussians potential, is the input to a set of ML models that return the electron density^53^. This learned density is the input for independent ML predictions of molecular energies based on DFT or CC electronic structure calculations, or the difference between these energies, in order to correct the DFT energy (final term in Eq. (3)). **b** Calculated energies for CC (dark blue) and DFT (dark orange) for 102 sample geometries relative to the lowest training energy (top), along with the relative energy errors for DFT compared to CC for each conformer (bottom). Note that the DFT energy errors are not a simple function of the energy relative to the minimum energy geometry (see Supplementary Fig. 2), as short O–H bond lengths tend to be too high in energy and stretched bonds are overstabilized. **c** Average out-of-sample prediction errors for the different ML functionals compared to the reference *E*^CC^ energies. The MAE of the *E*^DFT^ energies w.r.t. *E*^CC^ is also shown as a dashed line. **d** The energy surface (in kcal mol^−1^) of symmetric water geometries for ${E}_{{\mathrm{ML}}}^{{\mathrm{DFT}}}$ (orange) and ${E}_{\Delta \,\text{-}\,{\mathrm{DFT}}}^{{\mathrm{CC}}}$ (blue) after applying the Δ-DFT  correction (bottom). For this figure, DFT calculations use the PBE functional, and CC calculations use CCSD(T) (see “Methods” for more details).

In this paper, we describe an approach for generating an ML framework that satisfies the criteria outlined above. The ML model employed in this work is kernel ridge regression (KRR), the basic principles of which in the construction of density functionals have been developed over several years^63–69^. In order to advance our ML framework^53^ to the prediction of coupled-cluster (CC) energies, as opposed to DFT energies, one need only recognize that the basic ML construction procedure is independent of the source of inputs. Therefore, one could readily imagine training the aforementioned maps on a set of CC densities and energies. In practice, however, few quantum chemistry packages yield the CC electron density, as it is not something that is needed to find a CC energy. Therefore, in order to avoid the need to compute a CC electron density, we show that the density-energy map can be constructed by considering the CC energy as a functional of a DFT density obtained within a standard approximation such as PBE, i.e., we regress the CC energy from the PBE density. The density is used as the aforementioned descriptor for a given potential and can additionally serve as an input for learning other properties as well. The ML algorithm then learns to predict the CC energy as a functional of the approximate ML-predicted (descriptor) density. Importantly, we find that it is roughly as easy to train a model that returns the CC energy from the DFT density as it is to train for the self-consistent DFT energy itself. We additionally find that the use of a crudely approximated density results in a reduction in accuracy (even for DFT energies), showing the importance of using accurate densities. Drawing on existing ML experience^70^, we further show that it is possible to learn the difference between a DFT and a CC energy as a functional of the input DFT densities. Importantly, this can be done with greater efficiency than learning either DFT or CC energies separately. Referring to this approach as Δ-DFT , we show that the error in the training curve for Δ-DFT  drops far faster than that for learning either the DFT or the CC energies themselves, indicating that the error in DFT is much more amenable to learning than the DFT energy itself. Moreover, by exploiting molecular point group symmetries, we drastically reduce the amount of training data needed to achieve quantum chemical accuracy (~1 kcal mol^−1^), allowing us to extract CC energies from standard DFT calculations, with essentially no additional cost (beyond the initial generation of training data). That is, we create a system-specific ML model capable of yielding CCSD(T) accuracy at the cost of a standard DFT calculation. A single water molecule (see Fig. 1a) is used as the first benchmark of the new scheme. We use the same PBE density as a functional of the potential as in ref. ^53^ but now with various ML maps of the energy as a functional of the density. While the DFT calculation loses accuracy rapidly when the molecule is either compressed or extended, Δ-DFT  corrects these errors. We then consider the examples of ethanol, benzene, and resorcinol, all of which contain greater internal flexibility. We discuss the issue of sampling input geometries using finite-temperature MD simulations, arguing that care must be taken when these configurations do not reflect the target CCSD(T) energy surface (see Fig. 1b as an illustration for water). Resorcinol is further used as an example of using the ML scheme to generate an ab initio MD trajectory on the predicted underlying CCSD(T) energy surface. Obtaining such a trajectory typically requires hundreds to thousands or tens of thousands of energy and force calculations, which would be prohibitive using explicit CCSD(T) calculations but is routine using the ML model. This example reveals the importance of having CCSD(T) accuracy to describe a conformational change for which DFT produces quantitatively incorrect barriers. Finally, we take a step toward creating a more general model capable of predicting CCSD(T) energies of a small set of similar, but not identical, molecules. Resorcinol, phenol, and benzene are finally used to create an ML functional capable of describing multiple molecules. Here, molecular point group symmetries are exploited to expand the training dataset, thereby reducing the number of explicit CCSD(T) calculations needed to obtain chemical accuracy.

## Results

### Theory

A central difficulty in quantum chemistry is the fundamental incompatibility of the formalisms of DFT and wave-function based ab initio methods such as CCSD(T). Both aim to deliver the ground-state energy of a molecule as a function of its nuclear coordinates. Ab initio methods directly solve the electronic Schrödinger equation, albeit in an approximate yet systematic and controllable fashion. KS-DFT, by contrast, buries all the quantum complexity into an unknown functional of the density, i.e., the exchange-correlation (XC) energy, which must be approximated^71,72^. A myriad of different forms for such KS-DFT approximations exist. Unfortunately, there is currently no practical route for converting an approximation in one formalism to an approximation in the other, as there is no simple mathematical route to coupling the two formalisms.

In this work, we leverage ML to bypass this difficulty, by correcting DFT energies to CCSD(T) energies. Routine DFT calculations use some approximate XC functional and solve the Kohn-Sham equations self-consistently. However, an alternative approach has long been considered (e.g., ref. ^73^), in which the exact energy, *E*, is found by correcting an approximate self-consistent DFT calculation:

1 $$E={E}^{{\mathrm{DFT}}}[{n}^{{\mathrm{DFT}}}]+\Delta E[{n}^{{\mathrm{DFT}}}],$$

where DFT denotes the approximate DFT calculation, and Δ*E*, evaluated on the approximate density, is defined, formally, such that *E* is the exact energy. This is not the functional of standard KS-DFT, but it still yields exact energies and can be a more practical alternative in which one solves the KS equations within that approximation but corrects the final energy by Δ*E*. If *n*^DFT^ is a highly accurate approximation, then Δ*E* should not differ much from the intrinsic error of the DFT XC approximation. Recently, several classes of DFT calculations have been improved by using densities that are not self-consistent^74,75^. Thus, regression of DFT densities to find CC energies can be considered a system-specific construction of *ΔE*[*n*^DFT^] of the same kind as the system-specific construction of the HK map^53^. This differs from a general purpose, explicit XC functional approximation in that (i) it might only be accurate for the systems for which it has been trained, (ii) it has no simple closed form, and (iii) its functional minimum yields only an approximate density. However, using the results from the Supplementary Discussion 2.1, one can, in principle, construct the exact density from a sequence of such calculations. To avoid confusion, we note that Δ-DFT has nothing in common with, e.g., Δ-SCF, a useful alternative to TDDFT for extracting excited state energies in DFT^76^.

### Coupled cluster accuracy from ML DFT

Details of our approach are found in the “Methods” section. In brief, the approach constitutes a realization of the part of the Hohenberg-Kohn theorem that establishes a one-to-one mapping between external potentials *v*(**r**) and ground-state densities *n*(**r**) for a specified number of electrons. This map is expressed through the functional relationship *n*[*v*](**r**). In practice, we expand the density in an orthonormal basis *ϕ*_*l*_(**r**) as ${n}_{{\mathrm{ML}}}[v]=\mathop{\sum }\nolimits_{l = 1}^{L}{u}_{\mathrm{ML}}^{(l)}[v]{\phi }_{l}({\bf{r}})$ and learn the set density expansion coefficients $\{{{\bf{u}}}_{{}_{{\mathrm{ML}}}}[v]\}$^53^ in order to construct a learned DFT density ${n}_{{\mathrm{ML}}}^{{\mathrm{DFT}}}({\bf{r}})$. As previously noted, KRR is employed here as the ML model. A second KRR model is then used to predict energies from a higher level of theory, in this case CC energies:

2 $${E}_{{\mathrm{ML}}}^{{\mathrm{CC}}}[{n}_{{\mathrm{ML}}}^{{\mathrm{DFT}}}]=\mathop{\sum }\limits_{i=1}^{M}{{{\alpha }}}_{i}k({{\bf{u}}}_{{\mathrm{ML}}}[v],{{\bf{u}}}_{{\mathrm{ML}}}[{v}_{i}]).$$

where *k*(**u**_ML_[*v*], **u**_ML_[*v*_*i*_]) is the kernel, and {**α**} are the coefficients learned in the second KRR model. This allows us to create ${E}_{{\mathrm{ML}}}^{{\mathrm{CC}}}[{n}_{{\mathrm{ML}}}^{{\mathrm{DFT}}}]$, the chemically accurate CC energy, as a functional of the learned DFT density. (This corresponds to learning *E*^DFT^ + Δ*E* in Eq. (1)).

In order to demonstrate the methodology behind the map in Eq. (1), we begin by describing the process of learning the CC energy directly via Eq. (2) based on a set of 102 random water geometries (Fig. 1b and Supplementary Fig. 1). Note that the mean absolute error (MAE) of DFT energies relative to the CC energies (relative to the lowest energy conformer in the training set) is 1.86 kcal mol^−1^, with maximum errors of more than 6 kcal mol^−1^. The performance of the ${E}_{{\mathrm{ML}}}^{{\mathrm{DFT}}}[{n}_{{\mathrm{ML}}}^{{\mathrm{DFT}}}]$ and ${E}_{{\mathrm{ML}}}^{{\mathrm{CC}}}[{n}_{{\mathrm{ML}}}^{{\mathrm{DFT}}}]$ models was evaluated for training subsets containing 10, 15, 20, 30, 40 or 50 geometries, while the test set consisted of 52 geometries (Fig. 1c). Due to the small size of the dataset, we used cross-validation to obtain more stable estimates for the prediction accuracy of the models^69^. Details of the evaluation procedure are provided in the “Methods” section. As expected, the accuracy of each model improves with increasing training set size, but the benefit of predicting CC energies compared to DFT energies is immediately obvious. For this dataset, the MAE of *E*^DFT^ relative to *E*^CC^ (used here as the ground truth) is reached by ${E}_{{\mathrm{ML}}}^{{\mathrm{DFT}}}[{n}_{{\mathrm{ML}}}^{{\mathrm{DFT}}}]$ with 40 training geometries. Quantum chemical accuracy of 1 kcal mol^−1^ is obtained using slightly fewer (30) samples for the energy functional ${E}_{{\mathrm{ML}}}^{{\mathrm{CC}}}[{n}_{{\mathrm{ML}}}^{{\mathrm{DFT}}}]$, and an improved MAE of 0.24 kcal mol^−1^ with 50 training samples. Once constructed, the time to evaluate *E*_ML_[*n*] is the same regardless of the energy on which it is trained (for a fixed amount of training data). There is a clear benefit of training the model on the more accurate CC energies as long as a good performance can be achieved with a small number of samples from the more computationally expensive method.

Standard semilocal density functionals such as PBE typically yield highly accurate densities near equilibrium, and errors in atomization energies are dominated by errors in the energy rather than the self-consistent density^77^. However, far from equilibrium, these self-consistent densities can differ substantially from the exact density. In such density-sensitive cases, the energy error can be substantially increased by the error in the self-consistent density, leading to many failures of standard functionals^78^. The need to find accurate densities is bypassed by the ML-CC energy map, as it learns accurate energies even as a functional of an inaccurate density, as in Eq. (1).

### Reducing the CC cost with Δ-DFT 

Inspired by the concept of delta learning^79^, we also propose a machine-learning framework that is able to leverage densities and energies from lower-level theories (e.g., DFT) to predict CC level energies. This is achieved by correcting DFT energies using delta learning, which we denote as Δ-DFT . Instead of predicting the CC energies directly using our machine-learning model, we can instead train a new map $\Delta {E}_{{\mathrm{ML}}}^{{\mathrm{CC}}-{\mathrm{DFT}}}[{n}_{{\mathrm{ML}}}^{{\mathrm{DFT}}}]$ that yields the error in a DFT calculation (relative to CC) for each geometry (i.e., the second term in Eq. (1)). We define the corresponding total energy as

3 $${E}_{\Delta \,\text{-}\,{\mathrm{DFT}}}^{{\mathrm{CC}}}[{n}_{{\mathrm{ML}}}^{{\mathrm{DFT}}}]={E}^{{\mathrm{DFT}}}[{n}^{{\mathrm{DFT}}}]+\Delta {E}_{{\mathrm{ML}}}^{{\mathrm{CC}}-{\mathrm{DFT}}}[{n}_{{\mathrm{ML}}}^{{\mathrm{DFT}}}].$$

Correcting the DFT energies in this way leads to a dramatic improvement in the model performance, as seen in Fig. 1c. Remarkably, with only 10 training samples, the MAE of this ${E}_{\Delta \,\text{-}\,{\mathrm{DFT}}}^{{\mathrm{CC}}}\ [{n}_{{\mathrm{ML}}}^{{\mathrm{DFT}}}]$ model is already lower than the error of ${E}_{{\mathrm{ML}}}^{{\mathrm{CC}}}[{n}_{{\mathrm{ML}}}^{{\mathrm{DFT}}}]$ trained with 50 samples; using 50 training samples reduces the MAE of the Δ-DFT  model to only 0.013 kcal mol^−1^. The Δ-DFT  correction is easier to learn than the energies themselves, as illustrated in Fig. 1d for symmetric water geometries that were not included in the previous dataset. Although the optimized geometry differs slightly between DFT and CC, the Δ-DFT  approach provides a smooth map between the two types of electronic structure calculations as a functional of the density. For the most extreme geometries, the model errors for Δ-DFT  are smaller than for the direct models (see Supplementary Fig. 3) and depend differently on the geometry, indicating that there is information contained in the density beyond that of the external nuclear potential. We note in passing that Δ-DFT  links a particular DFT calculation to a particular CC level of theory, rendering comparisons between models trained on different calculations invalid (see Supplementary Discussion 2.2). The comparison between the Δ-DFT  and total energy ML models is further explored with larger molecules in the subsequent sections.

### Δ-DFT  with molecular symmetries

The next molecule chosen to evaluate our ML model is ethanol using geometries and energies from the MD17 dataset^32,33^. This molecule has two types of geometric minima, for which the alcohol OH is either an *anti* or doubly degenerate *gauche* position; the freely rotating CH_3_ group introduces additional variability into these possible geometries. Supplementary Fig. 4 shows the atomic distributions of the ethanol dataset after alignment based on heavy atom positions. The fact that ethanol possesses internal flexibility and a larger number of degrees of freedom than water naturally renders the learning problem more difficult. Hence, we expect that a greater number of training samples is needed to achieve chemical accuracy for the range of thermally accessible geometries. The dataset contains 1000 training and 1000 test samples with both DFT and CC energies (see Supplementary Fig. 5). The ML-HK map automatically incorporates equivalence for each chemical element, but we can also exploit the mirror symmetry of the molecule by reflecting H atoms through the plane defined by the three heavy atoms, effectively doubling the size of the training set, as outlined in the “Methods” section. To differentiate the models trained on datasets augmented by these symmetries, we add an *s* in front of the machine-learning model (e.g., *s*ML). Table 1 shows the prediction accuracies of the various *s*ML models for ethanol compared to some other state-of-the-art ML methods for the same dataset. The prediction error for DFT and CC energies is roughly equal to that of other ML models trained only on energies.

**Table 1:** MAEs (kcal mol^−1^) of sML ethanol maps compared to other ML models using forces and energies.

ML method	${E}_{{\mathrm{sML}}}[{n}_{{\mathrm{sML}}}^{{\mathrm{DFT}}}]$	${E}_{{\mathrm{s}}\Delta \text{-}{\mathrm{DFT}}}\ [{n}_{{\mathrm{sML}}}^{{\mathrm{DFT}}}]$	*E*_SchNet_^12^	*E*_sGDML_^33^
DFT	0.99	n/a	0.08 (0.93^a^)	0.07
CC	1.10	0.09	n/d	0.05

*n/a* not applicable, *n/d* not determined.

^a^${E}_{\,\text{SchNet}\,}^{{\mathrm{DFT}}}$ trained on energies alone.

It is also important to note that using the ${E}_{{\mathrm{s}}\Delta \,\text{-}\,{\mathrm{DFT}}}^{{\mathrm{CC}}}[{n}_{{\mathrm{sML}}}^{{\mathrm{DFT}}}]$ functional to correct low-cost DFT energies achieves a MAE for CC energies comparable to those of the most accurate force-based models, (without incurring the cost of evaluating CC forces for each training point). We note that Δ-learning does not improve the energy prediction over a direct force-based sGDML model for CC energies (see Supplementary Table 1). The ${E}_{\Delta \,\text{-}\,{\mathrm{DFT}}}^{{\mathrm{CC}}}[{n}_{{\mathrm{ML}}}^{{\mathrm{DFT}}}]$ functional based only on the original 1000 training geometries has a MAE of 0.15 kcal mol^−1^ (see Supplementary Table 2), hence using the ethanol symmetry reduces the MAE of the ML model by half while requiring the same number of CC calculations.

### Molecule optimization using ML functionals

Neither the training nor test configurations from the MD17 dataset^32,33^ include the minimum energy conformers of ethanol. Using the ML models, we predicted the energy of the *anti* and *gauche* conformers optimized using MP2/6-31G^*^ and the electronic structure methods used to generate the energies for each model. Note that MP2 and PBE have *gauche* as the global minimum, but the CCSD(T) global minimum is *anti*. Although all training geometries have energies more than 4.5 kcal mol^−1^ higher than the global minimum, the ML models are able to predict the energies of the minima with errors below chemical accuracy (see Table 2).

**Table 2:** Energy errors (kcal mol^−1^) of the sML-HK maps for ethanol at conventionally optimized geometries.

	MP2 *anti*	MP2 *gauche*	DFT *anti*	DFT *gauche*	CC *anti*	CC *gauche*
${E}_{{\mathrm{sML}}}^{{\mathrm{DFT}}}[{n}_{{\mathrm{sML}}}^{{\mathrm{DFT}}}]$	0.22	0.44	0.30	0.55	0.04	0.58
${E}_{{\mathrm{sML}}}^{{\mathrm{CC}}}[{n}_{{\mathrm{sML}}}^{{\mathrm{DFT}}}]$	0.12	0.49	0.19	0.62	0.13	0.66
${E}_{{\mathrm{s}}\Delta \,\text{-}\,{\mathrm{DFT}}}^{{\mathrm{CC}}}\ [{n}_{{\mathrm{sML}}}^{{\mathrm{DFT}}}]$	0.06	0.01	0.06	0.02	0.01	0.01

**Fig. 2:**
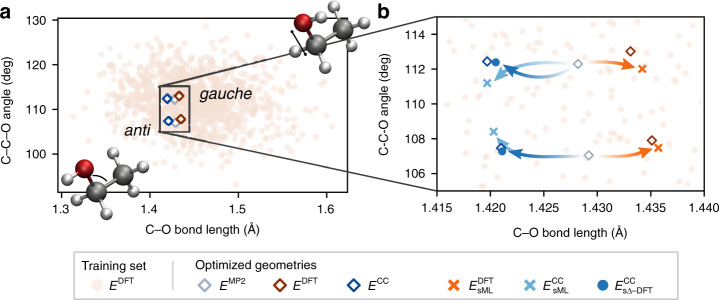
Molecular geometries of ethanol from the ML training set and optimizations. **a** 1000 unique configurations used for training (light orange circles), along with the *anti* and *gauche* minima optimized using conventional electronic structure methods (open diamonds). The distribution of *anti* and *gauche* conformers is shown in Supplementary Fig. 6. **b** The configurational space near the minima. Starting from MP2 geometries (*E*^MP2^, grey diamonds), the *E*_ML_-based optimizations reproduce the subtle differences in DFT- and CC-optimized geometries (dark orange and dark blue diamonds, respectively). For this figure, DFT calculations use the PBE+TS functional and CC calculations use CCSD(T) (see refs. ^32,33^ for more details).

In addition, the machine-learned energy function is sufficiently smooth to optimize ethanol using energy gradients computing from the ML model itself. Calculations for each conformer start from geometries optimized using MP2/6-31G^*^, which are slightly different from both DFT- and CC-optimized geometries. Figure 2b shows that despite the sparsity of training data near the minimum energy configurations, the ML models trained with different energies can differentiate between the DFT and CC minima with remarkable fidelity.

### ML model sensitivity to density inputs

Our results show that we can use ML models to map learned electron densities to several types of energy targets. This naturally raises the question of how sensitive our results are to the input density. If one does not need accurate self-consistent densities, why bother with the density at all? Why not, instead, simply learn the energy directly from the nuclear potential? To answer this, consider benzene and the 1500 geometries in the MD17 dataset^34^ (see Supplementary Figs. 7, 8). Due to benzene’s 24 point group (D_6*h*_) symmetries, applying our symmetrization approach on 1000 CC training points produces an effective dataset size of 24,000 geometries.

We first investigate the difference between *E*_sML_ models trained using the self-consistent DFT densities (*n*^DFT^) and those created by the ML-HK density map (${n}_{{\mathrm{sML}}}^{{\mathrm{DFT}}}$). Just as for ethanol, these models have accuracies comparable to other approaches that require CC forces for training (see Supplementary Table 3). Table 3 shows that for any of our energy functionals (${E}_{{\mathrm{sML}}}^{{\mathrm{DFT}}}$, ${E}_{{\mathrm{sML}}}^{{\mathrm{CC}}}$, or ${E}_{{\mathrm{s}}\Delta \,\text{-}\,{\mathrm{DFT}}}^{{\mathrm{CC}}}$), model performance differs negligibly when trained using these two-electron density representations because the density-driven errors of the ML-HK maps are small^53^. Relevant dimensionality estimation (RDE)^80^ quantifies the effective complexity that the ML models require for predicting, e.g., a particular set of energies given a set of densities (see Supplementary Tables 4, 5, 6). The direct *E*_sML_ models for benzene using the ground-state densities are all of similar complexity, with a comparable number of relevant data dimensions required to obtain similar accuracy. ${E}_{{\mathrm{s}}\Delta \,\text{-}\,{\mathrm{DFT}}}^{{\mathrm{CC}}}$ achieves higher accuracy with fewer relevant data dimensions than either direct model because the energy difference landscape is smoother and easier to learn.

**Table 3:** MAEs (kcal mol^−1^) for the MD17 benzene test set for the different density inputs and energy labels.

Density/energy	${E}_{{\rm{sML}}}^{{\rm{DFT}}}$	${E}_{{\rm{sML}}}^{{\rm{CC}}}$	$${E}_{{\rm{s}}\Delta \,\text{-}\,{\rm{DFT}}}^{{\rm{CC}}}\ $$	${E}_{{\rm{sML}}}^{{\rm{SAD}}}$	$${E}_{{\rm{s}}\Delta \,\text{-}\,{\rm{SAD}}}^{{\rm{CC}}}\ $$
*n*^DFT^	0.02	0.03	0.01	n/d	n/d
${n}_{{\rm{sML}}}^{{\rm{DFT}}}$	0.02	0.03	0.01	n/d	n/d
*n*^SAD^	0.03	0.08	0.06	0.22	0.22

*n/d* not determined

Next, we consider model performance when the molecular electron density is approximated by a superposition of atomic densities (SAD), which are conceptually similar to the pseudodensities used in other ML models^9,15^ and effectively translate the nuclear potential into electron densities, albeit without a proper description of the chemical bonds. While such densities (denoted as *n*^SAD^) cost little to generate, Table 3 shows that ML models trained on these inputs have errors that are at least twice those of models using more accurate densities. The RDE analysis shows that models based on *n*^SAD^ have comparable dimensionality for direct energy models but significantly lower signal-to-noise ratios (defined in SI for RDE analysis, Supplementary Eq. 2), thus rendering the energy models less accurate. Nonetheless, given the ever-present trade-off between accuracy and computational cost, SAD densities may be useful to avoid self-consistent optimization of the electron density for each geometry. In the case of SAD inputs, energy labels for the ML models would reflect the DFT functional evaluated on the approximate density (e.g., *E*^SAD^). For benzene, results are poorer for both the direct ML energy model (${E}_{{\mathrm{sML}}}^{{\mathrm{SAD}}}$) and Δ-DFT  (${E}_{{\mathrm{s}}\Delta \,\text{-}\,{\mathrm{SAD}}}^{{\mathrm{CC}}}$), although they are still within chemical accuracy. We understand the larger errors to be due to the increased variance of *E*^SAD^ labels (seven times that of the self-consistent dataset—see Supplementary Fig. 9) as well as their overall lower signal-to-noise ratio, as evidenced by the RDE analysis (see Supplementary Table 6).

The results presented thus far demonstrate that reasonably accurate ML models can be created using approximate densities that are inconsistent with the energy targets. Such ML models can be generated for applications where speed is more important than accuracy, for example, in the first few cycles of an active learning scheme^17^, where a cheap approximate density provides sufficient information to train models that ultimately would return CC energies with chemical accuracy. Finally, using accurate self-consistent densities as input significantly improves model performance for the same training and test geometries. These findings provide clear evidence that the electron density contains highly useful machine-learnable information about the molecular system beyond that contained in atomic positions alone.

### MD using CC energies

The final molecular example of 1,3-benzenediol (resorcinol) illustrates the utility of learning multiple ML functionals for the same system. Combining the ${E}_{{\mathrm{sML}}}^{{\mathrm{CC}}}[{n}_{{\mathrm{sML}}}^{{\mathrm{DFT}}}]$ with the more expensive and accurate ${E}_{{\mathrm{s}}\Delta \,\text{-}\,{\mathrm{DFT}}}^{{\mathrm{CC}}}\ [{n}_{{\mathrm{sML}}}^{{\mathrm{DFT}}}]$ method, we demonstrate how to run self-consistent MD simulations that can be used to explore the configurational phase space based on CC energies.

Resorcinol has two rotatable OH groups, two molecular symmetry operations, and more degrees of freedom than water, ethanol, or benzene, making this a more stringent test of the ML functionals. The initial datasets are generated from 1 ns classical MD simulations at 500 K and 300 K for the training and test sets, respectively (details are found in the “Methods” section). For the density representation, the 1000 conformer training set is augmented with the two symmetries, resulting in an effective training set size of 4000 samples (see Supplementary Fig. 10). The molecular geometries in the MD-generated training set have energies between 7 and 50 kcal mol^−1^ above the equilibrium conformer (as shown in Supplementary Fig. 11); the four local minima are also included in the dataset using geometries from MP2/6-31G^*^ optimizations, leading to 1004 unique training geometries and a total effective training set size of 4004 samples. These local minima, which differ in the orientation of the two alcohol groups, are separated by a rotational barrier of  ~ 4 kcal mol^−1^ (see Supplementary Fig. 12). The maximum relative energy errors between the DFT and the (ground truth) CC energies are 6.1 and 6.7 kcal mol^−1^, respectively, for geometries included in the training and test sets.

As with the other examples, ML model performance improves with increasing training set size (see Supplementary Fig. 13). When trained on 1004 unique training geometries (4004 training points), the MAE of predicted energies is around 1.3 kcal mol^−1^ for both ${E}_{{\mathrm{sML}}}^{{\mathrm{DFT}}}[{n}_{{\mathrm{sML}}}^{{\mathrm{DFT}}}]$ and ${E}_{{\mathrm{sML}}}^{{\mathrm{CC}}}[{n}_{{\mathrm{sML}}}^{{\mathrm{DFT}}}]$, and the error, when using ${E}_{{\mathrm{s}}\Delta \,\text{-}\,{\mathrm{DFT}}}^{{\mathrm{CC}}}\ [{n}_{{\mathrm{sML}}}^{{\mathrm{DFT}}}]$, is only 0.11 kcal mol^−1^. The *Δ*-DFT  accuracy is insensitive to the use of the ML-HK map for the density input, as shown in Supplementary Table 7, and is sufficient to run an MD simulation based on CC energies without the need of CC forces.

Although DFT energies may be sufficient for some molecules, the ability to use CC energies to determine the equilibrium geometries and thermal fluctuations is a promising advance. For resorcinol, the relative DFT energies can differ significantly from the CC energies, particularly near the OH rotational barrier that separates conformers (see Supplementary Fig. 12). Conformational changes are also rare events in the MD trajectories, making it crucial to describe the transitions accurately. For example, the exploration of the OH dihedral angles over a 10 ps MD trajectory from a DFT-based constant-temperature simulation at 350 K is shown in Supplementary Fig. 14. In this simulation, only one conformational change is observed, despite several excursions away from the local minima.

Using the ${E}_{{\mathrm{s}}\Delta \,\text{-}\,{\mathrm{DFT}}}^{{\mathrm{CC}}}$ approach, we could easily correct energies after running a conventional DFT-MD simulation. However, as shown in Supplementary Fig. 15, for snapshots along a 1.5 ps constant-energy simulation starting from a point near a conformer change, the MAE of DFT energies compared to CC energies for each snapshot is 1.0 kcal mol^−1^, with a maximum of just under 4.5 kcal mol^−1^. Therefore, a more promising use of the ML functionals is to run MD simulations using the CC energy function directly. An example ${E}_{{\mathrm{s}}\Delta \,\text{-}\,{\mathrm{DFT}}}^{{\mathrm{CC}}}\ [{n}_{{\mathrm{sML}}}^{{\mathrm{DFT}}}]$ trajectory starting from a random training point is shown in Supplementary Fig. 16, with an MAE of 0.2 kcal mol^−1^.

**Fig. 3:**
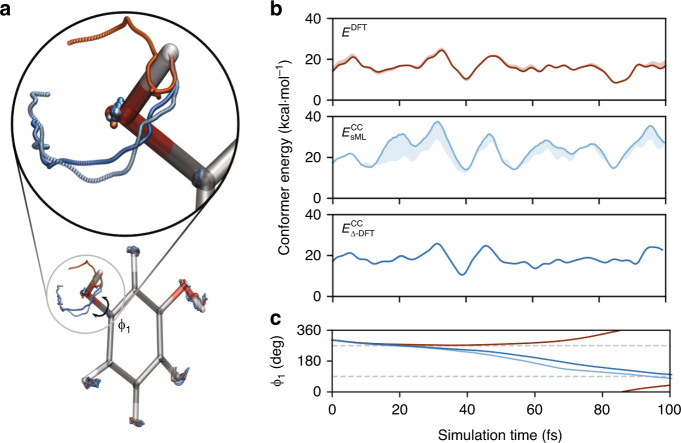
Resorcinol dynamics from an initial condition near a conformational change. **a** The atomic positions explored during 100 fs NVE MD trajectories run with standard DFT (dark orange), ${E}_{{\mathrm{sML}}}^{{\mathrm{CC}}}[{n}_{{\mathrm{sML}}}^{{\mathrm{DFT}}}]$ with RESPA-corrected forces (light blue), and ${E}_{{\mathrm{s}}\Delta \,\text{-}\,{\mathrm{DFT}}}^{{\mathrm{CC}}}\ [{n}_{{\mathrm{sML}}}^{{\mathrm{DFT}}}]$ (blue). **b** The conformer energy along each trajectory (solid lines), with the error relative to CC shown as a shaded line width. **c** The evolution of the C–C–O–H dihedral angle for each trajectory with dashed grey lines indicating the barrier between conformers. For this figure, all DFT calculations use PBE and all CC energies are from CCSD(T).

Starting from a different point in the DFT-generated trajectory serves to illustrate the importance of generating MD trajectories directly on the CC energy surface. As seen in Fig. 3, for constant-energy simulations starting from the same initial condition, a DFT-based trajectory does not have sufficient kinetic energy to traverse the rotational barrier, while the conformer switch does occur for the CC-based trajectory. Astonishingly, the ${E}_{{\mathrm{s}}\Delta \,\text{-}\,{\mathrm{DFT}}}^{{\mathrm{CC}}}\ [{n}_{{\mathrm{sML}}}^{{\mathrm{DFT}}}]$ trajectory has a MAE of only 0.18 kcal mol^−1^ relative to the true CC energies over a range of more than 15 kcal mol^−1^.

As the Δ-DFT  method requires performing a DFT calculation at each step of the trajectory, we can overcome this computational cost by combining the ML models. The middle panel of Fig. 3b shows the CC trajectory using a reversible reference-system based multi-time-step integrator^81^ to evaluate energies and forces primarily with the ${E}_{{\mathrm{sML}}}^{{\mathrm{CC}}}[{n}_{{\mathrm{sML}}}^{{\mathrm{DFT}}}]$ model as a reference and with periodic force corrections based on the more accurate ${E}_{{\mathrm{s}}\Delta \,\text{-}\,{\mathrm{DFT}}}^{{\mathrm{CC}}}\ [{n}_{{\mathrm{sML}}}^{{\mathrm{DFT}}}]$ every three steps (see Supplementary Note 1.4 and Supplementary Fig. 17 for more details). The resulting trajectory has a MAE of 3.8 kcal mol^−1^ relative to the true CC energies, with the largest errors in regions that are sparsely represented in the training set. This self-consistent exploration of the configurational space with the combined ML models provides an opportunity to improve the sampling in a cost-effective manner.

### Combining densities for improved sampling

The electron density provides some advantages as a descriptor of a chemical system over inputs that rely solely on local atomic environments or connectivity^11,12,82^. For a given periodic cell and number of basis functions, the same density input structure is able to describe systems with different numbers, types, and orders of atoms. In contrast, models that rely on an atomistic decomposition of the energy must have representations for the environment of each separate element (for example, see refs. ^6,26^). To improve the sampling represented in the training set for resorcinol, we can leverage overlap with configurational spaces sampled by similar, yet smaller and less costly, molecules. For example, adding data for phenol can provide better sampling of the rotation of an OH group, while the dynamics of benzene contains extensive sampling of C–C bonds.

To demonstrate this feature of density-based ML models, we use 1001 geometries for each of these two molecules as input configurations (see Supplementary Figs. 8, 18), along with the 1004 resorcinol configurations. We trained a set of density-to-energy maps, combining the symmetrized datasets, pairwise and as a complete set, and then we used the resorcinol test set to evaluate the performance of this model. In each case, the density-to-energy map was learned by combining the densities of the different molecules into a single dataset. The models using combinations of true or independently learned densities, displayed in Tables 4 and 5 and Supplementary Tables 8 and 9, show significant improvements in performance, with the prediction error being reduced by 30–60%. The results for models trained on DFT energies are similar to those for CC energies and can be found in Supplementary Table 10.

**Table 4:** ${E}_{{\rm{sML}}}^{{\rm{CC}}}$ MAEs (kcal mol^−1^) for combinations of molecular datasets evaluated on the resorcinol test set.

	Resorcinol	Resorcinol phenol	Resorcinol phenol benzene
*n*^DFT^	0.99	0.49	0.53
${n}_{{\rm{sML}}}^{{\rm{DFT}}}$	1.37	1.04	0.70
${n}_{{\rm{sML}}-{\rm{c}}}^{{\rm{DFT}}}$	n/a	0.69	0.71

*n/a* not applicable

**Table 5:** $${E}_{{\rm{s}}\Delta \,\text{-}\,{\rm{DFT}}}^{{\rm{CC}}}\ $$ MAEs (kcal mol^−1^) for combinations of molecular datasets evaluated on the resorcinol test set.

	Resorcinol	Resorcinol phenol	Resorcinol phenol benzene
*n*^DFT^	0.11	0.06	0.07
${n}_{{\rm{sML}}}^{{\rm{DFT}}}$	0.11	0.09	0.08
${n}_{{\rm{sML}}-{\rm{c}}}^{{\rm{DFT}}}$	n/a	0.07	0.09

*n/a* not applicable

In addition, we can analogously train an ML-HK map by combining the artificial potentials of the different molecules into one dataset in order to produce a combined map (${n}_{{\mathrm{sML}}-{\mathrm{c}}}^{{\mathrm{DFT}}}$). Using the combination of symmetrized phenol and resorcinol data to train the ML-HK map improves the performance of the direct ML energy models, although the Δ-DFT  approach is again less sensitive to the density representation. We note that, unlike the models with independently learned densities, simply adding more training data by including benzene in the ML-HK map, does not significantly change the results. Molecular similarity clearly affects the combination of ML-HK maps (see Supplementary Table 9 for resorcinol and benzene), but the ML density functionals are less sensitive and show improvement for all molecular combinations. We view this as a stepping stone toward learning a truly transferable model capable of predicting both densities and energies for a wide range of configurations and molecules.

## Discussion

DFT is used in at least 30,000 scientific papers each year^83^, and because of its low cost relative to wave function based ab initio methods, it can be used to compute energies of large molecules. Moreover, if geometry optimizations or MD simulations are desired, these would be beyond the reach of CCSD(T) level calculations owing to the high computational cost. However, if CCSD(T) is affordable for a small number of carefully chosen configurations, then our methodology provides one possible bridge between the DFT and CCSD(T) levels of theory.

There are two distinct modes in which our results can be applied. With Δ-DFT, the cost of a gas-phase MD simulation is essentially that of the DFT-based MD with a given approximate functional, plus the cost of evaluating a few dozen CCSD(T) energies. While the optimal selection of training points is an open question in the field of machine learning, the *Δ*-DFT  approach presented here may help to reduce the number of points necessary by learning an inherently smoother energy correction map. We stress that no forces are needed for training, making training set generation cheaper than other methods with similar performance. Compared to other machine-learning models, Δ-DFT  is well behaved and stable outside of the training set, since the zero-mean prior allows it to fall back on DFT results when far from the training set. The combination of Δ-DFT  with the ML models for DFT energies of ref. ^53^ yields both the efficiency from bypassing the KS equations and the accuracy of CCSD(T). While this yields accurate energy functions within the training manifold, it occasionally yields inaccurate energy gradients or forces in an MD simulation, which can be corrected with the Δ-DFT  forces using the appropriate integrators, as shown above.

Clearly, our methodology can be applied to any gas-phase MD simulation or geometry optimization for which CCSD(T) calculations can be performed for a reasonable number of carefully selected configurations. Gas-phase MD, for example, has many applications. Earlier studies focused on comparing equilibrium properties from simulations excluding or including (via the Feynman path integral) nuclear quantum effects^84–88^. More recent studies have focused on accurate spectroscopy and exploration of reactivity in small complexes and clusters^89–92^. For geometry optimization at the CCSD(T) level or testing of DFT energetics against CCSD(T) energies, DFT geometries often must be used due to the prohibitive cost of finding an optimum CCSD(T) geometry. For molecules with many soft modes, finding the geometry can require hundreds of evaluations of energies and forces. Here, we have shown how relatively few energies are needed in Δ-DFT  to produce an accurate energy functional, suggesting the possibility of using Δ-DFT  to speed up such searches, producing CC geometries for molecules that were previously prohibitive. For larger molecules and/or molecules interacting with an environment, recent schemes that embed an ab initio core within a larger DFT calculation^93^ could also be treated by this method, especially if Δ-DFT  need only be applied to the ab initio portion of the calculation. With suitable training sets, the ML approaches presented here have the potential to enable MD simulations for each of these systems.

Standard electronic structure methods require users to choose between accuracy and computational cost for each application. The success of our new ML approach connecting DFT densities to CC energies provides a new framework and strategy for linking formerly inconsistent calculations to reduce the penalty of this tradeoff. We have also demonstrated that the densities from a simpler molecule can be combined with a more complex system to improve the coverage of critical degrees of freedom. This promising result indicates that the smart use of combined densities from smaller molecular fragments could yield more accurate energies at even lower cost. Given that the CC-DFT energy difference landscape does not resemble the intrinsic energy landscapes of either of the underlying electronic structure methods, themselves, we hope future work will further explore this dissimilarity as a function of training set size and composition for Δ-DFT  models.

ML represents an entirely new approach to extracting energies from DFT calculations, avoiding some of the biases built into human-designed functionals, while also bypassing the need for strict self-consistency between the electron density and the resulting energy when an approximate result is sufficient. As shown here, ML provides a natural framework for incorporating results from more accurate electronic structure methods, thus bridging the gap between the CC and the DFT worlds while maintaining the versatility of DFT to describe electronic properties beyond energy and forces such as the dipole moment, molecular polarizability, NMR chemical shifts, etc. Along with these insights, the long and successful history of KS-DFT suggests that using the density as a descriptor may thus prove to be an excellent strategy for improved simulations in the future.

## Methods

### Machine-learning model

In order to predict the total energy of a system given only the *N*^a^ atomic positions of a molecule and using the electron density as a key descriptor, we can use the ML-HK map introduced in ref. ^53^, with the entire procedure being illustrated in Fig. 1a. Initially, we characterize the Hamiltonian by the external nuclear potential *v*(**r**), which we approximate using a sum of Gaussians as^94^

4 $$v({\bf{r}})=\mathop{\sum }\limits_{\alpha =1}^{{N}^{\text{a}}}{Z}_{\alpha }\exp \left(\frac{-| | {\bf{r}}-{{\bf{R}}}_{\alpha }| {| }^{2}}{2{\gamma }^{2}}\right),$$

where **r** are the coordinates of a spatial grid, **R**_*α*_ is a vector containing the atom coordinates of atom *α*, and *Z*_*α*_ is the nuclear charges of atom *α*. Finally, *γ* is a width hyperparameter. This Gaussian potential is then evaluated on a 3D grid around the molecule and used as a descriptor for the ML-HK model. For each molecule, cross-validation is used to determine the width parameter, *γ*, and the grid spacing for discretization of the associated Gaussian potential.

After obtaining the Gaussian potential, we use a KRR model to learn the approximate DFT valence electron density. In order to simplify the learning problem and avoid representing the density on a 3D grid, we expand the density map in an orthonormal basis set, and consequently learn the basis coefficients instead of the density grid points:

5 $${n}_{{\mathrm{ML}}}[v]({\bf{r}})=\mathop{\sum }\nolimits_{l = 1}^{L}{u}_{\mathrm{ML}}^{(l)}[v]{\phi }_{l}({\bf{r}}).$$

where *ϕ*_*l*_(**r**) is a basis function. In this work, a Fourier basis is employed. In the applications presented in this work, 12,500 basis functions (25 per dimension) proved sufficient for good performance. Use of KRR to learn these basis coefficients makes the problem more tractable for 3D densities, and more importantly, the orthogonality of the basis functions allows us to learn the individual coefficients independently:

6 $${u}_{{\mathrm{ML}}}^{(l)}[v]=\mathop{\sum }\nolimits_{i = 1}^{M}{\beta }_{i}^{(l)}k[v,{v}_{i}],$$

where *β*^(*l*)^ are the KRR coefficients and *k* is a kernel functional.

The independent and direct prediction of the basis coefficients makes the ML-HK map more efficient and easier to scale to larger molecules, since the complexity only depends on the number of basis functions. In addition, we can use the predicted basis coefficients to reconstruct the continuous density at any point in space, making the predicted density independent of a fixed grid and enabling computations such as numerical integrals to be performed at an arbitrary accuracy.

As a final step, another KRR model is used to learn the total energy from the density basis coefficients:

7 $${E}_{{\mathrm{ML}}}[{n}_{{\mathrm{ML}}}]=\mathop{\sum }\limits_{i=1}^{M}{{{\alpha }}}_{i}k({{\bf{u}}}_{{\mathrm{ML}}}[v],{{\bf{u}}}_{{\mathrm{ML}}}[{v}_{i}]),$$

where *k* is the Gaussian kernel.

### Exploiting point group symmetries

Training datasets for our machine-learning model can be easily enriched using the point group symmetries. To extract the point group symmetries and the corresponding transformation matrices we used the SYVA software package^95^. Consequently, we can multiply the size of the training set by the number of point group symmetries without performing any additional quantum chemical calculations simply by applying the point group transformations on our existing data.

### Cross-validation and hyperparameter optimization

Due to the small number of training and test samples, when evaluating the models on the water dataset, the data were shuffled 40 times, and for each shuffle a subset of 50 geometries was selected as the training set, with the remaining 52 being used as the out-of-sample test set. For the smaller training sets, a subset of the 50 training geometries was selected using *k*-means sampling.

The hyperparameters for all models were tuned using fivefold cross-validation on the training set. For the ML-HK map from potentials to densities, the following three hyperparameters were optimized individually for each dataset: the width parameter of the Gaussian potential *γ*, the spacing of the grid on which Gaussian potential is evaluated, and the width parameter *σ* of the Gaussian kernel *k*[**v**, **v**_*i*_]. For each subsequent density to energy map ${E}_{{\mathrm{ML}}}^{* }[n]$, only the width parameter of the Gaussian kernel *k*(**u**_ML_[**v**], **u**_ML_[**v**_*i*_]) needs to be chosen using cross-validation. Specific values are reported in the Supplementary Tables 11–15.

### Classical molecular dynamics

Training and test set geometries for resorcinol (1,3-benzenediol) and phenol were selected from a 1 ns trajectory generated via classical MD using the GAFF force field^96^. The local minima were optimized using MP2/6-31g^*^ in Gaussian09^97^. Symmetric atomic charge assignments were determined from a RESP fit^98^ to the HF/6-31g^*^ calculations, using the three distinct geometries with Boltzmann weights determined by the relative MP2 energies for resorcinol. All other standard GAFF parameters^96^ for the MD simulations were assigned using the AmberTools package^99^. To generate resorcinol and phenol conformers, classical MD simulations in a canonical ensemble were run at 300 K and 500 K using the PINY_MD package^100^ with massive Nosé-Hoover chain (NHC) thermostats^101^ for atomic degrees of freedom (length = 4, *τ* = 20 fs, Suzuki-Yoshida order = 7, multiple time step = 4) and a time step of 1 fs.

For the resorcinol and phenol training sets, we selected 1000 conformers closest to *k*-means centers from the 1 ns classical MD trajectory run at 500 K. The test sets comprise 1000 randomly selected snapshots from the 1 ns 300 K classical MD simulations. Datasets are aligned by minimizing the root mean square deviation (RMSD) of carbon atoms to the global minimum energy conformer.

### DFT molecular dynamics

Born-Oppenheimer MD simulations of a resorcinol molecule in the gas phase were run using DFT in the QUICKSTEP package^102^ of CP2K v. 2.6.2^103^. The PBE XC functional^104^ was used to approximate exchange and correlation, and a mixed Gaussian/plane wave (GPW) basis-set scheme^105^ was employed with DZVP-MOLOPT-GTH (m-DZVP) basis sets^106^ paired with appropriate dual-space GTH pseudopotentials^107,108^. Wave functions were converged to 1E-7 Hartree using the orbital transformation method^109^ on a multiple grid (*n* = 5) with a cutoff of 900 Ry for the system in a cubic box (*L* = 20 bohr). For the constant-temperature simulation, a temperature of 350 K was maintained using massive NHC thermostats^101^ (length = 4, *τ* = 10 fs, Suzuki-Yoshida order = 7, multiple time step = 4) and a time step of 0.5 fs.

### ML molecular dynamics

We used the atomistic simulation environment^110^ with a 0.5 fs time-step to run MD with ML energies. For the constant-temperature simulation, a temperature of 350 K maintained via a Langevin thermostat with a friction value of 0.01 atomic units (0.413 fs^−1^). Atomic forces were calculated using the finite difference method with *ϵ* = 0.001 Å.

### Electronic structure calculations

Optimizations for ethanol conformers were run using MP2/6-31g^*^ in Gaussian09^97^. DFT calculations for the ML models were run using Quantum ESPRESSO code^111^ with the PBE XC functional^104^ and projector-augmented wave approach^112,113^ with Troullier-Martin pseudopotentials replacing explicit ionic core electrons^114^. Molecules were simulated in a cubic box (*L* = 20 bohr) with a wave function cutoff of 90 Ry. The valence electron densities were evaluated on a grid with 125 points in each dimension. All CC calculations were run using Orca^115^ with CCSD(T)/aug-cc-pVTZ^116^ for water or CCSD(T)/cc-pVDZ^116^ for resorcinol and phenol.

## Supplementary information

Supplementary Information

## Data Availability

The data generated and used in this study are available at quantum-machine.org/datasets.

## Code availability

The code generated and used for this study is available at https://github.com/MihailBogojeski/ml-dft.
