# Supplementary material for: Quantum chemical accuracy from density functional approximations via machine learning
Source: Nat Commun. 2020 Oct 16;11:5223. doi: 10.1038/s41467-020-19093-1 (PMC7567867; doi:10.1038/s41467-020-19093-1)
Supplement: Supplementary file 1 — Supplementary Information [file 41467_2020_19093_MOESM1_ESM.pdf]

Supplementary Information:
Quantum chemical accuracy from density functional approximations via machine learning

Bogojeski, *et al.*

1 Supplementary Notes

1.1 Water dataset

The water molecule has three internal degrees of freedom, two O-H bonds and the H-O-H angle, with bond lengths of 0.97 Å and a bond angle of 104.0° (1.82 radians) for the geometry optimized with the PBE functional. A preliminary dataset composed of 1000 molecular geometries was generated by uniformly sampling O-H bond lengths and H-O-H angles. The 102 geometries for the training set were selected based on the minimum energy conformer of the 1000 structures (1.6 kcal·mol^{-1} above the optimized geometry), with bond lengths in the range 0.97 ± 0.25 Å and the bond angle in the range of 115 ± 26° (2.00 ± 0.45 radians). In order to simplify the learning problem, the molecules were aligned in the xy-plane with the bisector of the H-O-H angle along the y-axis and the longer O-H bond in the same quadrant. The resulting geometries are shown in Supplementary Fig. 1.

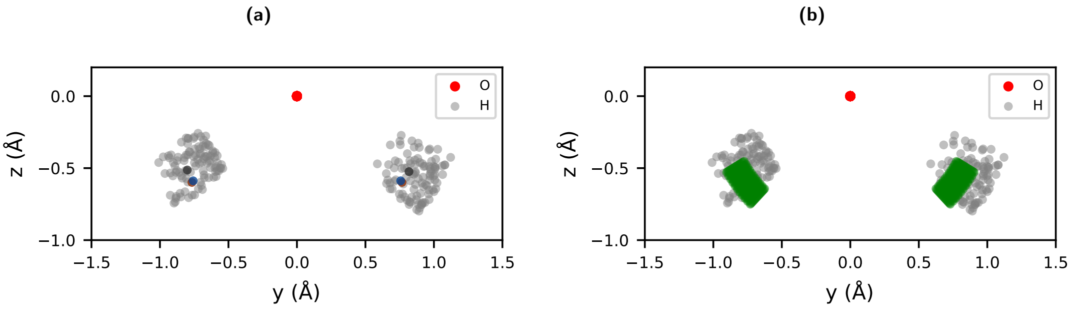

Supplementary Figure 1: Distribution of 102 water molecule geometries included in the dataset, with bond lengths in the range of 0.72 to 1.22 Å and bond angles in the range of 93.4 to 140.7° (1.63 to 2.46 radians). a) Darker circles indicate the minimum energy conformer of the training set (black) and the geometries after optimization with PBE (orange) or CCSD(T) (blue), b) The extent of the symmetric water test set is shown in green with bond lengths in the range of 0.90 to 1.05 Å and bond angles in the range of 88.5 to 119.5° (1.54 to 2.08 radians).

The conventional coupled cluster calculations were run in Molpro Quantum Chemistry Software [1] or Orca v.3.0.3 [2] using CCSD(T)/aug-cc-pVTZ [3]. For all water geometries in this work, using a single reference wave function is appropriate (T1 diagnostic < 0.02).

The model is trained on total calculated energies, but there is a shift in the energy range due to the differing bonding energy between electronic structure methods. Therefore, we report relative energies in Fig. 1b based on the lowest energy in the grand training set. These relative energy errors are also shown in Supplementary Fig. 2 and are used to calculate the MAE reported in Fig. 1c. The potential energy surfaces in Fig. 1d are shown relative to the lowest energy conformer for each energy method.

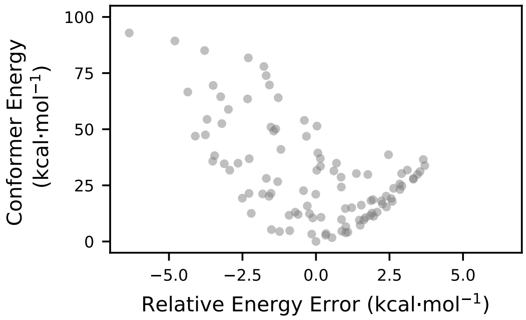

Supplementary Figure 2: Relative energy errors (CC-DFT) for the 102 water geometries in the grand training set plotted against the relative energy as calculated using CC.

The symmetric water dataset was constructed for the range of bond lengths and angles sampled during a DFT-MD simulation at 300 K using deviations of 3σ from the average value. The resulting bond range is 0.97 $\pm$ 0.075 Å and the angle range is 104.0 $\pm$ 15.5° (1.81 $\pm$ 0.27 radians). For the symmetric water conformers, the errors for predicted relative energies are qualitatively and quantitatively different for the direct ML energy functionals and the Δ-DFT approach, as shown in Supplementary Fig. 3. The errors in the direct methods reflect the overlap between the training set geometries and the out-of-sample test set, with the largest errors for small angles on the edge of the training set (coordinate range shown in Supplementary Fig. 1). The Δ-DFT errors are significantly smaller and qualitatively different, as they depend much more strongly on the bond length than the bond angle.

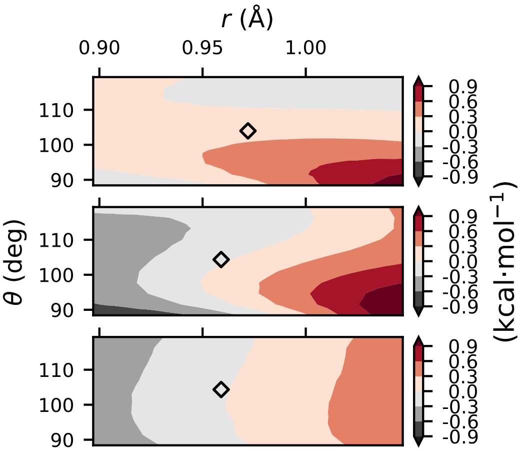

Supplementary Figure 3: The relative energy errors for symmetric water geometries compared to the values calculated using traditional electronic structure methods using $E^{\mathrm{DFT}}_{\mathrm{ML}}[n^{\mathrm{DFT}}_{\mathrm{ML}}]$ (top, PBE), $E^{\mathrm{CC}}_{\mathrm{ML}}[n^{\mathrm{DFT}}_{\mathrm{ML}}]$ (middle, CCSD(T)), and $E^{\mathrm{CC}}_{\Delta\text{-}\mathrm{DFT}}[n^{\mathrm{DFT}}_{\mathrm{ML}}]$ (bottom, CCSD(T)). The optimized geometries are indicated by open diamonds for each method.

1.2 Ethanol dataset

Training and test set data for ethanol is from the MD17 dataset [4, 5]. The 1000 unique geometries in the training set are aligned with the three heavy atoms defining a reflection plane for the symmetry-augmented dataset. Supplementary Fig. 4 shows the atomic distributions of the ethanol datasets after alignment and inclusion of symmetry-generated training points.

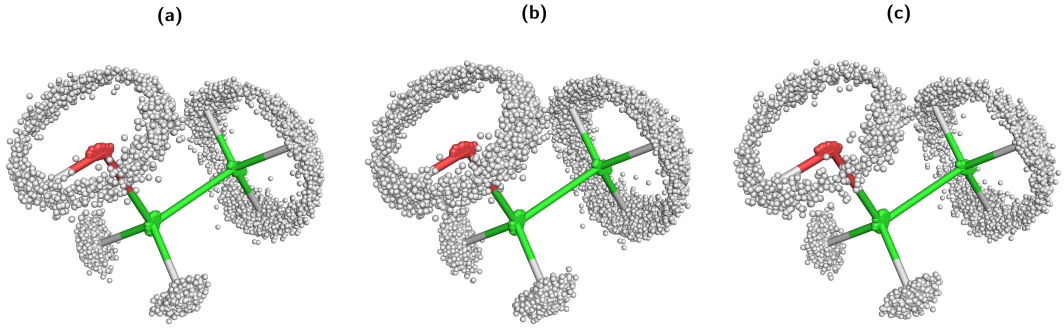

Supplementary Figure 4: Distribution of ethanol geometries included in the dataset, including geometries of a) the original 1000 training set, b) the effective 2000 point training set generated using mirror symmetry, and c) the 1000 point test set.

The specific data generation methods are reported in Refs. [4, 5]; Briefly, the conformers were generated using DFT-MD at 500 K (PBE+TS) and the coupled cluster energies use CCSD(T)/cc-pVTZ. For all ethanol coupled cluster calculations, the T1 diagnostic is below 0.02. In addition to DFT- and CC-optimized structures, we also optimize each local minima using MP2/6-31g* in Gaussian09 [6]. All geometries are similar, with the largest difference seen for the C-O bond length (DFT > MP2 > CC), as shown in Fig. 2. We report relative energies for ethanol based on the lowest energy minima for each electronic structure method (*anti* for CC by 0.08 kcal·mol^{-1} and *gauche* for DFT by 0.10 kcal·mol^{-1}). These relative energy errors are shown in Supplementary Fig. 5, with maximum relative energy errors of 5.6 kcal·mol^{-1} and 5.9 kcal·mol^{-1} for geometries included in the training and test sets, respectively.

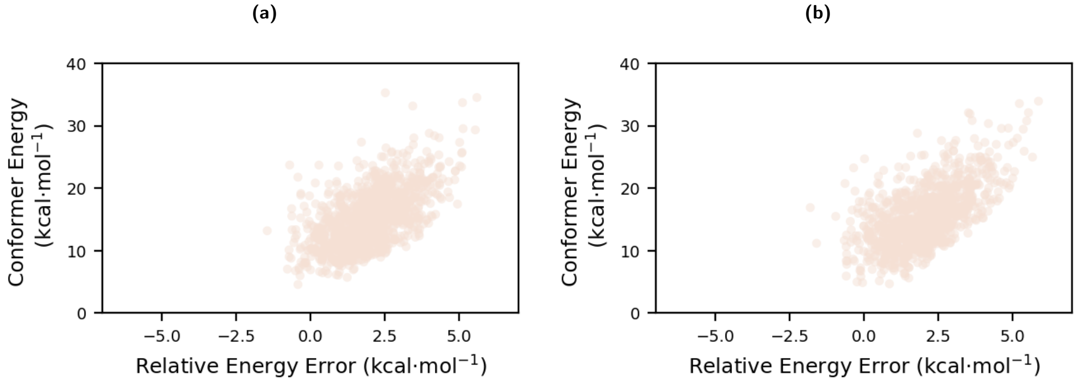

Supplementary Figure 5: Relative energy errors (CC-DFT) plotted against the relative conformer energy calculated using CC for a) the 1000 unique ethanol geometries in the training set and b) the 1000 ethanol geometries in the test set.

The local minima of ethanol differ most clearly in the dihedral angle of the alcohol OH, but the

optimized geometries also differ in the C-C-O angle. The training set shown in Fig. 2a is also shown in Supplementary Fig. 6 sorted by OH dihedral angle.

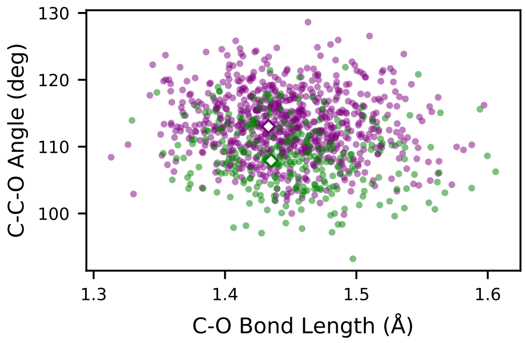

Supplementary Figure 6: Ethanol training geometries sorted by conformer so that all structures with the alcohol OH in the *anti* (*gauche*) basin are shown in green (purple). The open diamonds show the location of the corresponding DFT-optimized geometries.

Using Δ-DFT with force-based methods. Δ-learning is a universal approach that can be applied with any machine learning model to improve the accuracy, however, some models stand to benefit more from Δ-learning than others. To explore this we took sGDML [5], one of the state-of-the-art force-field based ML models, and trained it on the differences between the CC and DFT forces of 1000 ethanol geometries. Table 1 compares the accuracies of our density-based model and sGDML when predicting DFT, CC and Δ-DFT energies. We notice that Δ-learning with sGDML shows essentially no difference from the originally reported sGDML results for directly learning CC energies, while our model improves by a factor of 10 using the Δ-DFT approach.

Supplementary Table 1: Comparison of the MAEs (kcal·mol^{-1}) of density-based models and sGDML for ethanol trained on 1000 samples for different energy targets, showing the minimal improvement for sGDML using Δ-DFT compared to the models proposed in this work.

ML Model	$E_{\mathrm{sML}}[n_{\mathrm{ML}}^{\mathrm{DFT}}]$	sGDML [5]
DFT	0.99	0.072
CC	1.10	0.052
Δ-DFT	0.09	0.049

A likely reason for this is that sGDML uses forces for training, which contain more information than just the energy. This means that the prediction accuracy for sGDML converges more quickly, so it cannot benefit from Δ-learning as much.

Using Δ-DFT with molecular symmetry.

Supplementary Table 2: MAEs (kcal·mol^{-1}) of the ML maps for ethanol trained on 1000 samples, with and without augmenting the dataset using symmetry operations.

Dataset/Model	$E_{\mathrm{ML}}^{\mathrm{DFT}}[n_{\mathrm{ML}}^{\mathrm{DFT}}]$	$E_{\mathrm{ML}}^{\mathrm{CC}}[n_{\mathrm{ML}}^{\mathrm{DFT}}]$	$E_{\Delta\text{-DFT}}^{\mathrm{CC}}[n_{\mathrm{ML}}^{\mathrm{DFT}}]$
With symmetries	0.99	1.10	0.09
No symmetries	1.73	1.92	0.15

1.3 Benzene dataset

Training and test set data for benzene is from the MD17 dataset [4, 5], with the addition of the geometry optimized using MP2/6-31g* in Gaussian09 [6]. The specific data generation methods are reported in Refs. [4, 5]. Briefly, the conformers were generated using DFT-MD at 500 K (PBE+TS) and the coupled cluster energies use CCSD(T)/cc-pVDZ. To normalize the molecular positions, the optimized geometry is aligned with all C atoms in the xy-plane, and two atoms on the y-axis. The 1000 unique geometries in the MD17 training set are aligned by minimizing the root mean squared deviation of C atoms ($RMSD_C$) from the optimized structure, with an $RMSD_C$ of 0.045 Å for the training data and 0.046 Å for the test data. Benzene is a highly symmetric molecule, and using our symmetrization approach increases the effective dataset size by a factor of 24. Supplementary Fig. 7 shows the atomic distributions of the benzene datasets after alignment and inclusion of symmetry-generated training points.

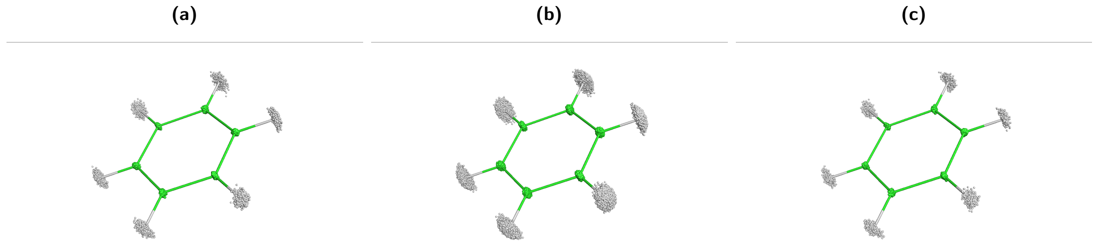

Supplementary Figure 7: Distribution of benzene geometries included in the MD17 dataset, including geometries of a) the original 1001 point training set, b) the 24,001 point effective training set generated using symmetry operations, and c) the 500 point test set.

For each dataset and electronic structure method (including the ML models), we report energies relative to the optimized MP2/6-31g* geometry (included in the training set). The relative energy errors using the conventional DFT method are shown in Supplementary Fig. 8, with maximum relative energy errors of 3.2 kcal·mol^{-1} and 3.0 kcal·mol^{-1} for geometries included in the training and test sets, respectively. We note that for all benzene coupled cluster calculations, the T1 diagnostic is below 0.02, so a single reference method is appropriate.

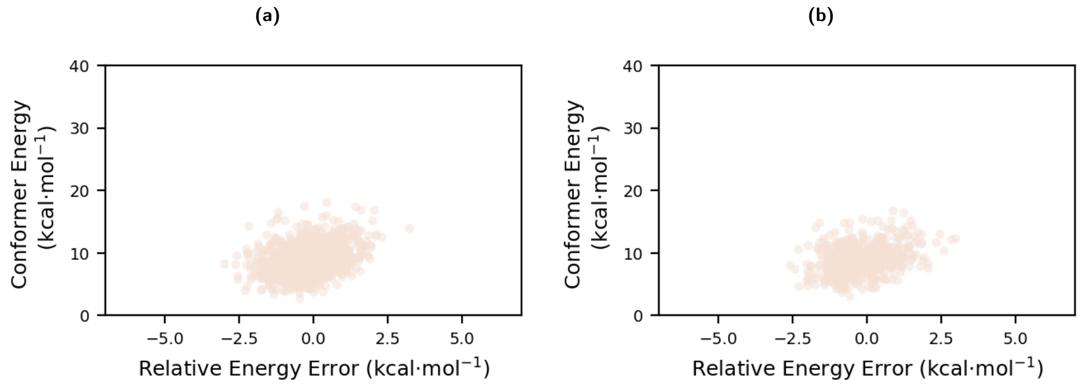

Supplementary Figure 8: Relative energy errors (CC-DFT) plotted against the relative conformer energy calculated using CC for a) the 1000 unique benzene geometries in the training set and b) the 500 benzene geometries in the test set.

Supplementary Table 3: MAEs (kcal·mol^{-1}) of the sML maps for benzene trained on 1000 MD17 samples, compared to other published state-of-the-art ML models that use forces in addition to energies.

ML method	$E_{\mathrm{sML}}[n^{\mathrm{DFT}}_{\mathrm{sML}}]$	$E_{\mathrm{s\Delta\text{-}DFT}}[n^{\mathrm{DFT}}_{\mathrm{sML}}]$	E_{SchNet} [7]	E_{sGDML} [5]
DFT	0.02	n/a	0.08 (1.19^{a})	0.10
CC	0.03	0.01	n/d	0.004

^{a} $E^{\mathrm{DFT}}_{\mathrm{SchNet}}$ trained on energies alone; n/a = not applicable, n/d = not determined.

In addition to the self-consistent densities from fully converged DFT calculations, we also generate input electron densities from a standard initial guess in electronic structure calculations: the superposition of atomic densities (SAD). The energy returned by the DFT functional prior to any density optimization steps is $E^{\mathrm{DFT}}[n^{\mathrm{SAD}}]$. Supplementary Fig. 9 shows the relative energy errors compared to the CC values for each conformer, using E^{SAD} of the MP2/6-31g* optimized geometry as the reference. The E^{SAD} range is considerably larger than for the converged calculations, with a variance that is seven times larger and a qualitatively different distribution of energy errors.

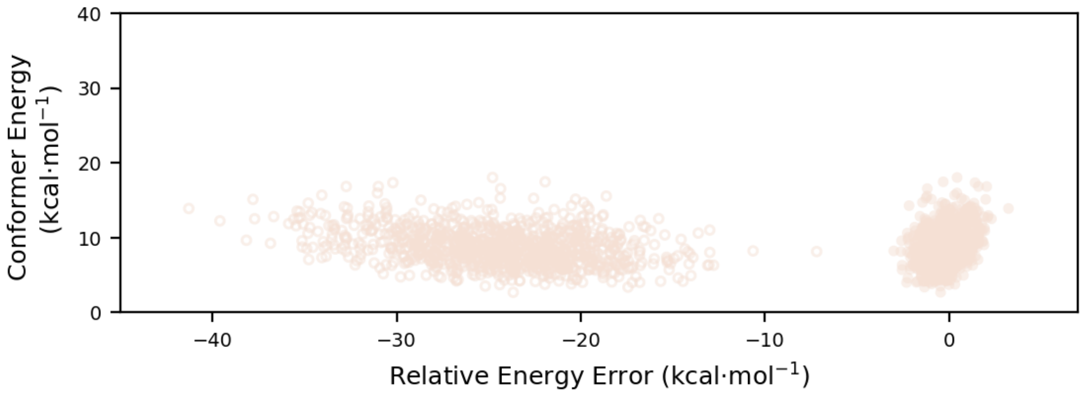

Supplementary Figure 9: Relative energy errors (CC-DFT) plotted against the relative conformer energy calculated using CC for the MD17 training data evaluated using superpositions of atomic densities (open circles) and converged densities (filled circles, same data as in Supplementary Fig. 8a).

Relevant dimensionality estimation of different density types. To further analyze the effect of using different types of densities as input descriptors, we used relevant dimensionality estimation (RDE) [8]. With kernel based methods, the relevant information for a supervised learning problem is contained up to a negligible error in the feature subspace defined by a leading number of components given by kernel principal component analysis (PCA) [9]. RDE allows us to estimate the dimensionality of the subspace containing the label-relevant information, thereby giving us a way to quantify the effective complexity that each of the ML models requires to predict the energy labels using different types of density descriptors.

To obtain the RDE of a kernel ridge regression model, we perform kernel PCA [9] in the feature space of the kernel, where we obtain the kernel PCA components $\mathbf{c}_1, \ldots, \mathbf{c}_M$ as the eigenvectors of the kernel matrix. Subsequently, we determine the number of label relevant dimensions d in the feature space, which is the number of leading kernel PCA components needed to accurately reconstruct the labels. The labels are reconstructed by projecting them on the leading d kernel PCA components: $\hat{\mathbf{Y}}(d) = \sum_{i=1}^{d} \mathbf{c}_i \mathbf{c}_i^T \mathbf{Y}$. In our case labels denote the respective energies (CC or PBE).

Finally, number of relevant dimensions d is estimated by minimizing the following objective

function [8]:

$$\mathcal{L}(d) = \frac{d}{M}\log(\sigma_1^2) + \frac{M-d}{M}\log(\sigma_2^2)$$
$$\text{where} \quad s_i = \mathbf{c}_i^T\mathbf{Y}, \quad \sigma_1^2 = \frac{1}{d}\sum_{i=1}^{d} s_i^2 \quad \text{and} \quad \frac{1}{M-d}\sum_{i=d+1}^{M} s_i^2. \tag{1}$$

Given an optimized number of relevant dimensions d, we can estimate the noise level of the labels as the error of the reconstructed labels $\hat{\mathbf{Y}}(d)$ with respect to the true labels $\mathbf{Y}$. Additionally, we can estimate the signal-to-noise ratio as:

$$SNR(d) = \frac{\mathrm{Var}(\hat{\mathbf{Y}}(d))}{\mathrm{Var}(\hat{\mathbf{Y}}(d) - \mathbf{Y})}. \tag{2}$$

The resulting estimates for the relevant dimensions, noise level, and signal-to-noise ratio obtained by applying RDE to the different ML models are shown in Supplementary Tables 4, 5, 6.

Supplementary Table 4: Estimated relevant dimensions for the different density and energy types used for the benzene.

Density/Energy	$E_{\mathrm{sML}}^{\mathrm{DFT}}$	$E_{\mathrm{sML}}^{\mathrm{CC}}$	$E_{\mathrm{s\Delta\text{-}DFT}}^{\mathrm{CC}}$	$E_{\mathrm{sML}}^{\mathrm{SAD}}$	$E_{\mathrm{s\Delta\text{-}SAD}}^{\mathrm{CC}}$
n^{DFT}	3432	3475	2632	n/d	n/d
$n_{\mathrm{sML}}^{\mathrm{DFT}}$	3413	3452	2061	n/d	n/d
n^{SAD}	3436	3436	3251	2647	2021

n/d = not determined

Supplementary Table 5: Estimated label noise level based on the estimated relevant dimensions for the different density and energy types used for the benzene.

Density/Energy	$E_{\mathrm{sML}}^{\mathrm{DFT}}$	$E_{\mathrm{sML}}^{\mathrm{CC}}$	$E_{\mathrm{s\Delta\text{-}DFT}}^{\mathrm{CC}}$	$E_{\mathrm{sML}}^{\mathrm{SAD}}$	$E_{\mathrm{s\Delta\text{-}SAD}}^{\mathrm{CC}}$
n^{DFT}	0.05	0.05	0.01	n/d	n/d
$n_{\mathrm{sML}}^{\mathrm{DFT}}$	0.05	0.06	0.01	n/d	n/d
n^{SAD}	0.07	0.08	0.01	0.15	0.16

n/d = not determined

Supplementary Table 6: Estimated label signal-to-noise ratio based on the estimated relevant dimensions for the different density and energy types used for the benzene.

Density/Energy	$E_{\mathrm{sML}}^{\mathrm{DFT}}$	$E_{\mathrm{sML}}^{\mathrm{CC}}$	$E_{\mathrm{s\Delta\text{-}DFT}}^{\mathrm{CC}}$	$E_{\mathrm{sML}}^{\mathrm{SAD}}$	$E_{\mathrm{s\Delta\text{-}SAD}}^{\mathrm{CC}}$
n^{DFT}	1196.56	1208.05	4335.93	n/d	n/d
$n_{\mathrm{sML}}^{\mathrm{DFT}}$	1162.68	1161.95	4010.18	n/d	n/d
n^{SAD}	661.39	623.38	3639.97	677.66	374.98

n/d = not determined

1.4 Resorcinol dataset

In order to construct the ML functionals, the resorcinol datasets were generated using standard classical MD simulations. To sample more extreme geometries, training set points were selected from a 500 K trajectory, while the test set was taken from a 300 K simulation. After aligning the molecules to have all C atoms in one plane, $RMSD_C$ is 0.045 Å for the 300 K dataset and 0.053 Å for the 500 K dataset. Supplementary Fig. 10 shows the geometries and C-C-O-H dihedral angles in the original training set, the training set with symmetry operations applied, and the test set. The finite temperature classical MD simulations primarily sample geometries around the minimum energies, but all conformers are more than 7 kcal·mol^{-1} higher in energy than the global minimum. The all-electron CCSD(T)/cc-pVDZ [3] calculations were run using Orca v.3.0.3 [2], and the single reference wave functions have a maximum T1 diagnostic of 0.012 for all conformers.

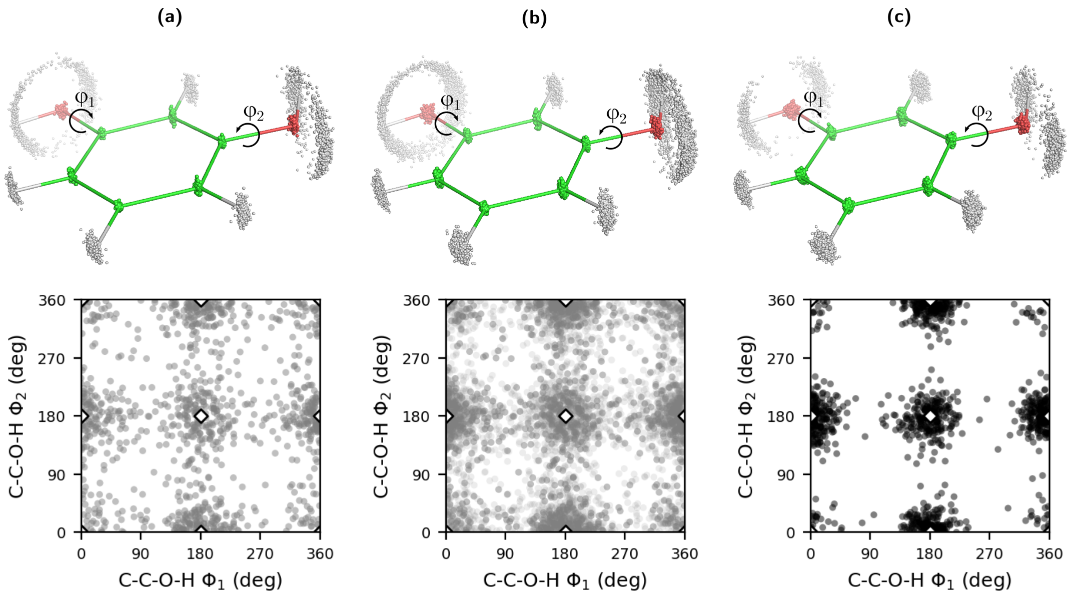

Supplementary Figure 10: Sampled geometries for resorcinol showing atom distribution (top) and distribution of the dihedral angle of the two -OH groups (bottom) for the 1004 point training set (a), 4004 point training set generated with molecular symmetry operations (b), and 1000 point test set (c). The minimum energy conformers are shown as sticks and open black diamonds.

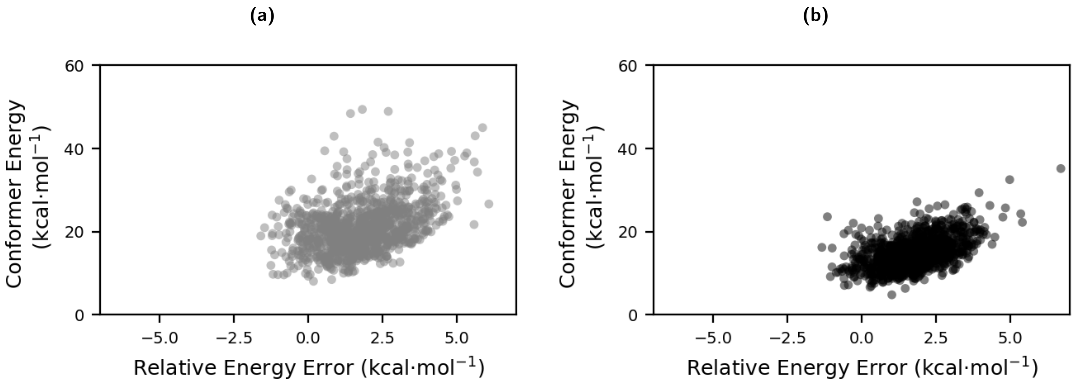

Supplementary Figure 11: Relative energy errors (CC-DFT) plotted against the relative conformer energy calculated using CC for a) the 1000 unique resorcinol geometries in the training set (additional four minima not shown) and b) the 1000 resorcinol geometries in the test set.

Resorcinol rotational barrier. The sparse sampling of geometries away from the minima is due to an energy barrier for each OH group rotation. For an optimized geometry, rotation of a single OH requires crossing an energy barrier of almost 4 kcal·mol^{-1} based on CC calculations, as shown in Supplementary Fig. 12. The barrier height based on PBE energies is even larger, but can successfully be corrected using the Δ-DFT approach.

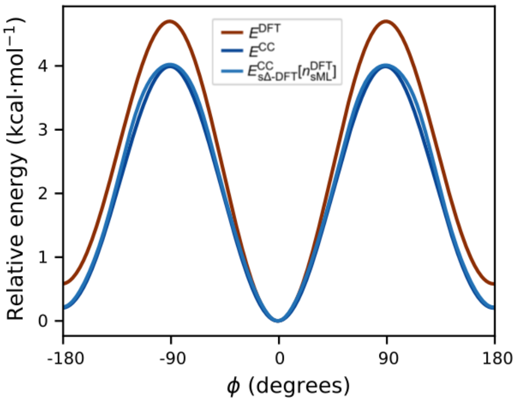

Supplementary Figure 12: Relative energies for DFT and CC showing the difference in the OH rotational barrier between the two methods, along with the energy predicted by the $E^{\mathrm{CC}}_{\mathrm{s\Delta\text{-}DFT}}[n^{\mathrm{DFT}}_{\mathrm{sML}}]$ energy map.

Resorcinol model performance. Supplementary Fig. 13 shows the MAE of the different ML models for different training set sizes of the resorcinol dataset. Each training set is chosen from the original 1000 points using k-means and then expanded using the four symmetry operations prior to training. The Δ-DFT model has an error below 1 kcal·mol^{-1} when using only 25 data points. In this manuscript, model performance results and MD simulations are run using the full 1004 point training set to facilitate comparison between the best direct energy models and the $E^{\mathrm{CC}}_{\mathrm{s\Delta\text{-}DFT}}$ approach.

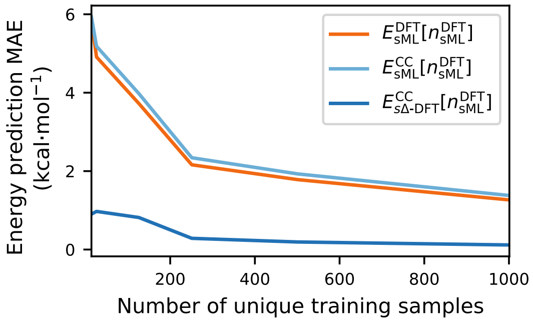

Supplementary Figure 13: Comparison of the out-of-sample prediction performance for the DFT, CC, and Δ-DFT energy prediction models for different training set sizes.

For resorcinol, using true densities rather than ML-HK densities as input improves the performance for the direct ML models more than in the case of benzene (see Supplementary Table 7). In contrast, the Δ-DFT model error is considerably smaller than either direct model and is unaffected by the accuracy of the electron density, indicating again that the Δ-DFT approach is robust and the energy difference landscape is smoother and easier to learn than the total energy itself.

Supplementary Table 7: MAEs (kcal·mol^{-1}) of the ML models trained on different electronic structure energies for densities learned by the ML-HK map and true densities as inputs.

Density/Energy	$E^{\mathrm{DFT}}_{\mathrm{sML}}$	$E^{\mathrm{CC}}_{\mathrm{sML}}$	$E^{\mathrm{CC}}_{\mathrm{s\Delta\text{-}DFT}}$
n^{DFT}	0.94	0.99	0.11
$n^{\mathrm{DFT}}_{\mathrm{sML}}$	1.26	1.37	0.11

Resorcinol DFT-based MD. For a DFT-based MD simulation, resorcinol samples two different conformational basins during a 10 ps NVT simulation at 350 K. The aligned atomic positions and C-C-O-H dihedral angles are shown in Supplementary Fig. 14.

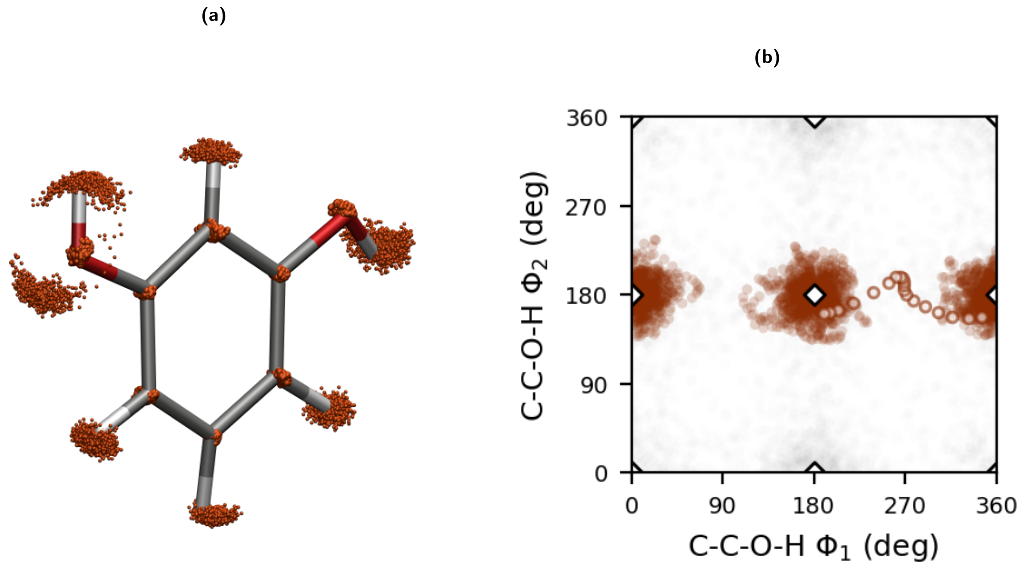

Supplementary Figure 14: DFT-based MD for resorcinol at 350 K samples two conformational basins with a) atomic coordinates after alignment for the 10 ps trajectory, and b) the C-C-O-H dihedral angles during the trajectory, with the portion of the trajectory that crosses the rotational energy barrier highlighted with open circles and local minima shown as open diamonds.

Starting from an initial condition close to the barrier crossing event, a DFT-based MD simulation was run using NVE for 1.5 ps. The relative CC energy for snapshots along this trajectory are shown in Supplementary Fig. 15 along with the relative energy errors of the DFT calculations themselves (relative to CC).

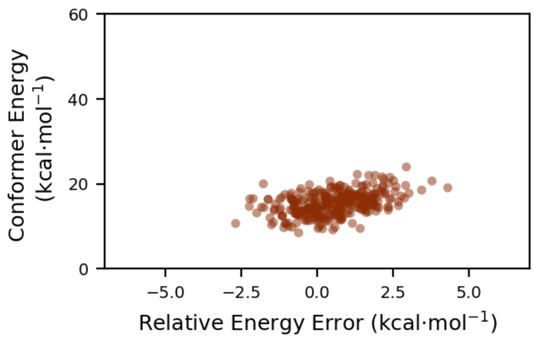

Supplementary Figure 15: Relative energy errors (CC-DFT) plotted against the relative conformer energy calculated using CC for an NVE DFT-based MD trajectory for resorcinol starting from an initial condition near the barrier crossing.

Resorcinol ML-MD. The $E^{\rm CC}_{\rm s\Delta\text{-}DFT}[n^{\rm DFT}_{\rm sML}]$ model can be used to generate a self-consistent MD trajectory, as seen in Supplementary Fig. 16. Starting from a random training point, the 150 fs trajectory has a MAE of only 0.2 kcal·mol^{-1} relative to the true CC energies. There is no drift in energy error as the trajectory proceeds, indicating that the Δ-DFT approach is stable for a range of conformers.

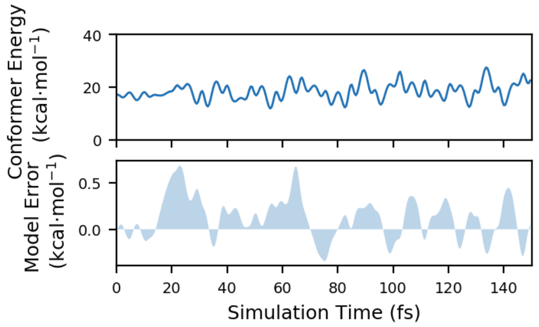

Supplementary Figure 16: $E^{\mathrm{CC}}_{\mathrm{s}\Delta\text{-DFT}}[n^{\mathrm{DFT}}_{\mathrm{sML}}]$ energy along the self-generated trajectory is shown in the top panel. The MAE relative to CC is 0.2 kcal·mol^{-1}, which is smaller than the linewidth, so the energy error is shown separately in the bottom panel.

Resorcinol multiple time-step MD. The $E^{\mathrm{CC}}_{\mathrm{sML}}[n^{\mathrm{DFT}}_{\mathrm{sML}}]$ model can also be used to generate a self-consistent MD trajectory. Starting from an initial condition (atomic positions and velocities) from the DFT-based NVT simulation, the $E^{\mathrm{CC}}_{\mathrm{sML}}[n^{\mathrm{DFT}}_{\mathrm{sML}}]$ trajectory explores a region of phase space outside of the training set, as seen in the error relative to CC shown in Supplementary Fig. 17a. The sparsity of training data near this initial position leads to uncertainty in the model. This issue can be mitigated by correcting the forces every few steps using the $E^{\mathrm{CC}}_{\mathrm{s}\Delta\text{-DFT}}[n^{\mathrm{DFT}}_{\mathrm{sML}}]$ model (see Supplementary Methods 3.4) to prevent excursions into high energy regions. Correcting the forces every fifth time step ($m = 5$) using the $E^{\mathrm{CC}}_{\mathrm{s}\Delta\text{-DFT}}[n^{\mathrm{DFT}}_{\mathrm{sML}}]$ model already improves the conformer sampling (Supplementary Fig. 17b), with a similar performance seen for a $m = 3$ trajectory (Supplementary Fig. 17c). The trajectories shown in Supplementary Fig. 17c and d are the same as in Fig. 3b.

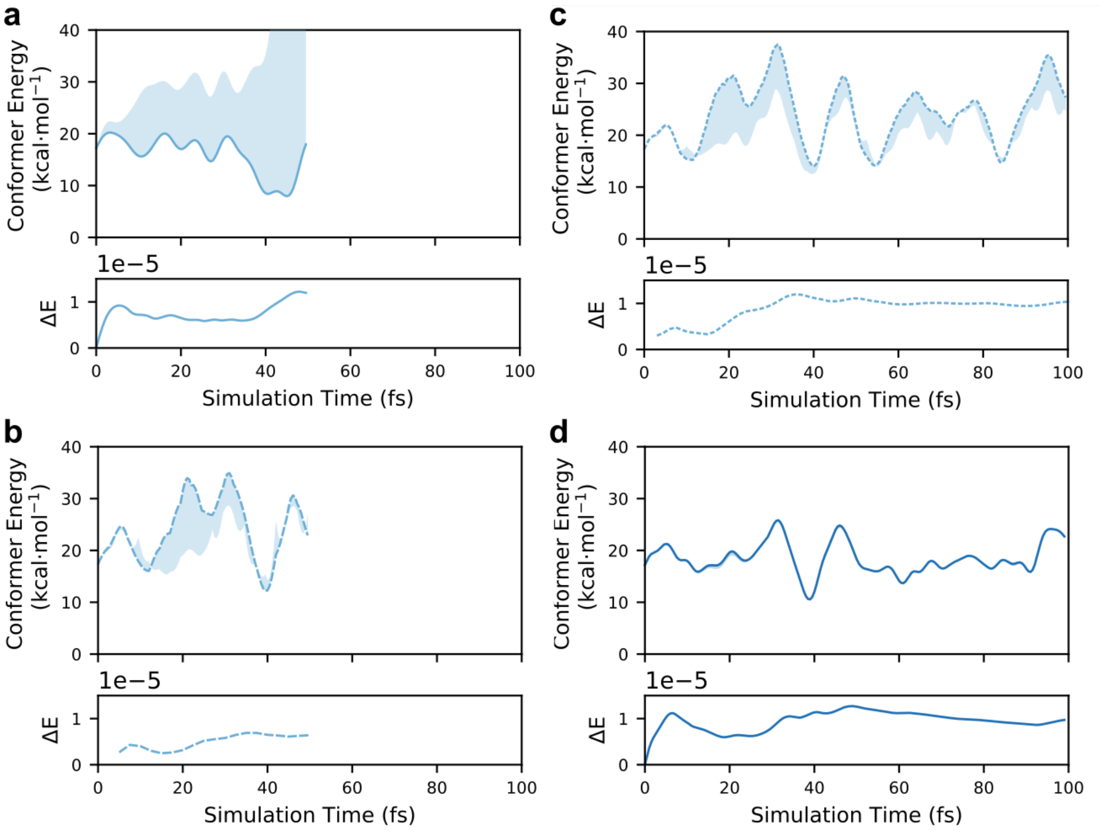

Supplementary Figure 17: For NVE trajectories starting from the same initial condition, $E^{\mathrm{CC}}_{\mathrm{sML}}$ trajectory energies are shown in the top panels (with errors relative to the true CC energies shown as shaded regions) and conserved quantities in the bottom panels for a) a simulation run with $E^{\mathrm{CC}}_{\mathrm{sML}}[n^{\mathrm{DFT}}_{\mathrm{sML}}]$ energies and forces, b) a $E^{\mathrm{CC}}_{\mathrm{sML}}[n^{\mathrm{DFT}}_{\mathrm{sML}}]$ simulation where the $E^{\mathrm{CC}}_{\mathrm{s\Delta\text{-}DFT}}[n^{\mathrm{DFT}}_{\mathrm{sML}}]$ is used to correct the forces every fifth time step, c) a $E^{\mathrm{CC}}_{\mathrm{sML}}[n^{\mathrm{DFT}}_{\mathrm{sML}}]$ simulation where the $E^{\mathrm{CC}}_{\mathrm{s\Delta\text{-}DFT}}[n^{\mathrm{DFT}}_{\mathrm{sML}}]$ is used to correct the forces every third time step, and d) a $E^{\mathrm{CC}}_{\mathrm{s\Delta\text{-}DFT}}[n^{\mathrm{DFT}}_{\mathrm{sML}}]$ simulation.

1.5 Phenol dataset

Phenol datasets were generated using standard classical MD simulations. As for resorcinol, training set points were selected from a 500 K trajectory, while the test set was taken from a 300 K simulation. After aligning the molecules based on C atom RMSD to the equilibrium geometry, $\mathrm{RMSD_C}$ is 0.043 Å for the 300 K dataset and 0.052 Å for the 500 K dataset. In the finite temperature classical MD simulations, all sampled conformers are higher in energy than the global minimum by more than 5 kcal·mol^{-1} for the 500 K training trajectory and more than 2 kcal·mol^{-1} for the 300 K test trajectory. The all-electron CCSD(T)/cc-pVDZ [3] calculations were run using Orca v.3.0.3 [2], and the single reference wave functions have a maximum T1 diagnostic of 0.010 for all conformers.

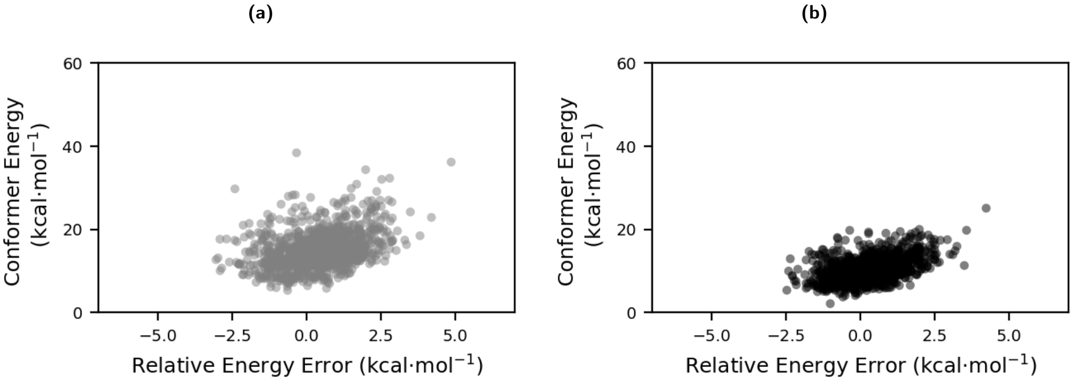

Supplementary Figure 18: Relative energy errors (CC-DFT) plotted against the relative conformer energy calculated using CC for a) the 1000 unique phenol geometries in the training set (minimum geometry not shown) and b) the 1000 phenol geometries in the test set.

1.6 Combining densities for improved sampling

Electron density datasets can include data from multiple molecules since the Fourier basis representation does not depend on the type, number, or order of atoms in a molecule of interest. As an example, we combine the previously discussed training data for benzene (from MD17 dataset [4, 5]) and resorcinol (from classical MD) with phenol (sampled from classical MD, see Methods section). The models are evaluated on the resorcinol test set.

When adding the phenol molecules to the resorcinol dataset, the molecule is aligned so that the OH group is in the same orientation as one of the OH groups of resorcinol. When symmetrizing the 1000 sampled geometries, we obtain an effective dataset of 4000 points. Supplementary Table 8 shows the performance for the combined model of resorcinol and phenol aligned with one OH group. Since resorcinol has more than one alcohol, we also generated an 8000 point training set by reflecting each symmetrized phenol molecule to align the OH with each of the two OH groups of resorcinol. Despite both models using the same 1000 energy labels, the doubled model shows improved performance and these results are reported in Tables 4, 5, and Supplementary Table 10.

Supplementary Table 8: MAEs (kcal·mol^{-1}) for the combined dataset of resorcinol and phenol (with one OH aligned) evaluated on the resorcinol test set.

Density/Energy	$E^{\mathrm{PBE}}_{\mathrm{sML}}$	$E^{\mathrm{CC}}_{\mathrm{sML}}$	$E^{\mathrm{CC}}_{\mathrm{s\Delta\text{-}DFT}}$
n^{DFT}	0.74	0.79	0.07
$n^{\mathrm{DFT}}_{\mathrm{sML}}$	1.10	1.15	0.09
$n^{\mathrm{DFT}}_{\mathrm{sML-c}}$	0.89	0.94	0.08

Supplementary Table 9: MAEs (kcal·mol^{-1}) for the combined dataset of resorcinol and benzene evaluated on the resorcinol test set.

Density/Energy	$E^{\mathrm{PBE}}_{\mathrm{sML}}$	$E^{\mathrm{CC}}_{\mathrm{sML}}$	$E^{\mathrm{CC}}_{\mathrm{s\Delta\text{-}DFT}}$
n^{DFT}	1.04	1.12	0.09
$n^{\mathrm{DFT}}_{\mathrm{sML}}$	1.10	1.17	0.11
$n^{\mathrm{DFT}}_{\mathrm{sML-c}}$	1.19	1.29	0.14

Supplementary Table 10: Comparison of the $E^{\mathrm{DFT}}_{\mathrm{sML}}$ MAEs (kcal·mol^{-1}) for the DFT energy models trained on different combinations of the resorcinol, phenol, and benzene datasets evaluated on the resorcinol test set.

	resorcinol	resorcinol phenol	resorcinol phenol benzene
n^{DFT}	0.94	0.49	0.45
$n^{\mathrm{DFT}}_{\mathrm{sML}}$	1.26	0.98	0.66
$n^{\mathrm{DFT}}_{\mathrm{sML-c}}$	n/a	0.65	0.68

2 Supplementary Discussion

2.1 Using non-self-consistent densities

Our model appears to violate one of the basic results of density functional theory, as it produces CCSD(T) energies from PBE densities. Hohenberg and Kohn [10] showed that, for any given approximate energy functional, one can minimize the energy functional to find a self-consistent formula for the density. The Kohn-Sham scheme is defined [11] to find that density when only the exchange-correlation contribution to the energy is approximated. The purpose of this section is show how the self-consistent density could be found, at least in principle, and also to argue that the energetic consequences of using the PBE density would be negligible here.

There is an exact formula for extracting the energy from any approximation for the ground-state energy for a given external potential [12]:

$$n(\mathbf{r}) = \frac{\delta E[v]}{\delta v(\mathbf{r})} \tag{3}$$

where $E[v]$ is the ground-state energy associated with one-body potential $v(\mathbf{r})$. This could be used to extract a density pointwise from any such approximation: Add a small narrow Gaussian centered at $\mathbf{r}_0$ to $v(\mathbf{r})$, and note the corresponding change in energy. In the limit of infinitely narrow, infinitely weak perturbations, this yields the density at $\mathbf{r}_0$. Of course, such a procedure is highly impractical in a standard basis set of atom-centered Gaussians, but could be easily employed to find specific moments of the density. If the perturbation is a weak static electric field, the prescription yields the dipole moment, as can be seen by multiplying both sides by $\mathbf{r}$ and integrating over all space.

Almost all electronic structure calculations in chemistry and materials science are aimed at finding accurate ground-state energies and the many properties that can be derived from them, such as geometries and barriers. The error in any DFT calculation can be split into two contributions, the functional error and a density-driven error (the energy error due to an incorrect density) [13]. In most DFT calculations (including all those given here), the self-consistent density is so accurate that the energy error is dominated by the functional error [14]: using the exact density in the approximate functional has negligible effect on the energy error. Recent arguments that attempt to distinguish the quality of functionals by constructing metrics of density errors [15] have not held up when analyzed in terms of energies [14].

We can use Supplementary Eq. (3) to analyze the present situation. We know it must be satisfied by the PBE density and energy functional. Thus the difference between the PBE and CCSD(T) densities is simply

$$\Delta n(\mathbf{r}) = \frac{\delta \Delta E^{\mathrm{PBE}}[v]}{\delta v(\mathbf{r})}. \tag{4}$$

This will be a very small energy for normal systems. The fact that the energy difference is easier to learn than the PBE energy itself suggests a smoothness of energy difference with respect to the potential, making density differences tiny.

We also note an additional twist on this question in the context of machine learning. Long ago, Görling and Levy [16] and others pointed out that one could define an exact energy functional on an approximate density, such as the HF density. In fact, as was noted by Li *et al.* [17], to learn accurate *energies*, a very crude representation of the density suffices, so long as it forms a sufficiently useful feature for the energy. In a prototype problem (particle in a box with potential well), with even a very small grid (far too coarse to find accurate solutions to the Schrödinger equation) and essentially exact energies, one could still use kernel ridge regression to find a highly accurate ML functional.

Thus use of PBE densities to find CCSD(T) energies is both practical and theoretically allowed and well understood. On the practical side, it completely avoids the need to extract CCSD(T)

densities to train upon. Because the density is not needed to perform a CCSD(T) calculation, it is not available from many CCSD(T) codes. On the theoretical side, we know (a) the errors in energies will be very small, (b) how to correct them if need be, and (c) that the kernel ridge regression has no difficulty learning on the PBE density. We note that the models using the SAD approximation perform reasonably well when assigned energy labels corresponding to the self-consistent PBE density. When using the PBE energies evaluated on the SAD density, the model errors are considerably worse, indicating that using a density that reflects the bonding environment is an important consideration if accurate energies are required.

On the other hand, use of non-self-consistent densities breaks the standard relation between energies and forces from the Hellmann-Feynman theorem, and small errors in energies do not automatically imply small errors in forces. Since we use numerical derivatives of energies throughout this paper, we extract the correct forces, but there will be corrections if analytical derivatives are attempted. Whether or not these are significant is beyond the scope of the present work.

2.2 Challenges when comparing combined models

The combined density model is a step towards a more universal machine learned density functional that can be used for CC level simulations across a wider range of molecules. Other published ML models aim to provide energies for many molecules, and it appears tempting to compare their performance to our density-based approach. For example, ANI-CC is a general neural network potential that has been trained on approximate CCSD(T) energies of a wide range of molecules [18]. However, a number of differences in model construction mean that a direct comparison of these energies with the current model is not one-to-one. As the ML methods are trained for different energy targets, we instead are required to use relative energies, with the global minimum geometry of resorcinol as the zero point.

When evaluated on our resorcinol test set, the ANI-CC model has a mean absolute difference (MAD) of 1.78 kcal·mol^{-1}. Comparing this error to the results in Table ??, we see that our combined model of resorcinol, phenol and benzene differs from ANI-CC by more than a factor of two when directly predicting the E^{CC}, and by more than a factor of 20 for the Δ-DFT variant.

This result was to be expected, since ANI-CC and our model differ in many respects, including the very energy targets to be returned and the training data selection. The density-based ML models have been trained specifically to reproduce the geometry fluctuations of a few related molecules, rather than including many functional groups. In the general, while the relative energies of both model types are strongly correlated, they still represent different training goals. Users of the Δ-DFT approach need to fully understand that the energy correction is trained to link a specific DFT calculation (functional and basis set) to a specific CC energy (type of excitations and basis set).

3 Supplementary Methods

3.1 Kernel ridge regression

Kernel ridge regression (KRR) [19] is a powerful machine learning method for non-linear regression. Non-linearity is achieved by incorporating the kernel trick into Kernel ridge regression, extending linear ridge regression, which finds the optimal linear mapping from the inputs to the labels under ℓ_2 regularization, by exploiting the kernel trick to map the inputs to a high-dimensional non-linear feature space. Let $\mathbf{x}_1, \ldots, \mathbf{x}_M \in \mathbb{R}^d$ be the training data points and let $\mathbf{Y} = [\mathbf{y}_1, \ldots, \mathbf{y}_M]^T$ be their respective labels. The KRR model for a new input sample $\mathbf{x}^*$ is then given by:

$$\mathbf{y}^* = \sum_{i=1}^{M} \alpha_j k(\mathbf{x}^*, \mathbf{x}_i), \tag{5}$$

where k is a kernel function and $\boldsymbol{\alpha} = [\alpha_1, \ldots, \alpha_M]^T$ are the model weights. The model weights are obtained by solving the following optimization problem:

$$\min_{\boldsymbol{\alpha}} \left\{ \sum_{i=1}^{m} \left| \mathbf{y}_i - \sum_{j=1}^{m} \alpha_j k(\mathbf{x}_i, \mathbf{x}_j) \right|^2 + \lambda \boldsymbol{\alpha} \mathbf{K} \boldsymbol{\alpha} \right\} \tag{6}$$

where λ is a regularization parameter and $\mathbf{K}$ is the kernel matrix with $\mathbf{K}_{ij} = k(\mathbf{x}_i, \mathbf{x}_j)$. The analytical solution to the minimization problem is then given by

$$\boldsymbol{\alpha} = (\mathbf{K} + \lambda \mathbf{I})^{-1} \mathbf{Y}. \tag{7}$$

In this paper we use the Gaussian (radial basis function) kernel

$$k(\mathbf{x}, \mathbf{x}') = \exp\left(-\frac{||\mathbf{x} - \mathbf{x}'||^2}{2\sigma^2} \right), \tag{8}$$

where the kernel width σ is a model parameter that needs to be tuned using cross-validation.

3.2 Decomposability of the ML-HK map

When using the ML-HK map to predict electron density, the contributions of the prediction error to the cost function are given by

$$\begin{aligned} err(\boldsymbol{\beta}) &= \sum_{i=1}^{M} ||n_i - n_{\mathrm{ML}}[\mathbf{v}_i]||^2_{\mathcal{L}_2} \\ &= \sum_{i=1}^{M} \left\| n_i - \sum_{l=1}^{L} u^{(l)}_{\mathrm{ML}}[\mathbf{v}_i] \phi_l \right\|^2_{\mathcal{L}_2} . \end{aligned} \tag{9}$$

By writing the density in terms of its basis representation and assuming orthogonality of the basis functions we obtain

$$
\begin{aligned}
err(\boldsymbol{\beta}) &= \sum_{i=1}^{M} \left\| \sum_{l=1}^{L} u_i^{(l)} \phi_l - \sum_{l=1}^{L} u_{\mathrm{ML}}^{(l)}[\mathbf{v}_i] \phi_l \right\|_{\mathcal{L}_2}^2 \\
&= \sum_{i=1}^{M} \left\| \sum_{l=1}^{L} \left(u_i^{(l)} - u_{\mathrm{ML}}^{(l)}[\mathbf{v}_i] \right) \phi_l \right\|_{\mathcal{L}_2}^2 \\
&= \sum_{i=1}^{M} \int \sum_{l=1}^{L} \left(u_i^{(l)} - u_{\mathrm{ML}}^{(l)}[\mathbf{v}_i] \right) \phi_l(\mathbf{r}) \sum_{l'=1}^{L} \left(u_i^{(l')} - u_{\mathrm{ML}}^{(l')}[\mathbf{v}_i] \right) \phi_{l'}^*(\mathbf{r}) d\mathbf{r} \\
&= \sum_{i=1}^{M} \sum_{l,l'=1}^{L} \left(u_i^{(l)} - u_{\mathrm{ML}}^{(l)}[\mathbf{v}_i] \right) \left(u_i^{(l')} - u_{\mathrm{ML}}^{(l')}[\mathbf{v}_i] \right) \int \phi_l(\mathbf{r}) \phi_{l'}^*(\mathbf{r}) d\mathbf{r} \\
&= \sum_{i=1}^{M} \sum_{l=1}^{L} \left(u_i^{(l)} - u_{\mathrm{ML}}^{(l)}[\mathbf{v}_i] \right)^2 \\
&= \sum_{i=1}^{M} \sum_{l=1}^{L} \left(u_i^{(l)} - \sum_{j=1}^{M} \beta_j^{(l)} k(\mathbf{v}_i, \mathbf{v}_j) \right)^2 .
\end{aligned}
\tag{10}
$$

The resulting equation shows that the error can be decomposed into the independent error contributions for each of the basis coefficients. By viewing the errors independently we obtain L separate KRR minimization problems, and analogously to equations 6 and 7 we obtain the analytical solutions

$$
\boldsymbol{\beta}^{(l)} = \left(\mathbf{K}_{\sigma^{(l)}} + \lambda^{(l)} \mathbf{I} \right)^{-1} \mathbf{u}^{(l)}, \quad l = 1, \ldots, L, \tag{11}
$$

where for each basis function ϕ_l, $\lambda^{(l)}$ is a regularization parameter, $\mathbf{u}^{(l)}$ is a vector containing the training set coefficients for the l-th basis function and $\mathbf{K}_{\sigma^{(l)}}$ is a Gaussian kernel matrix with width $\sigma^{(l)}$.

3.3 Cross-validation

All hyperparameters used in the model are estimated solely on the training set. The width γ and spacing Δ hyperparameters for the artificial Gaussians potential as well as the kernel width σ and the regularization parameter λ were optimized individually for each molecule. In both cases the hyperparameter optimization was performed using cross-validation [20] on the training set. After training and cross-validation, the model is fixed and is applied unchanged on the out-of-sample test set. The optimal hyperparameters for the artificial potentials grid selected using the cross-validation procedure are given in Supplementary Table 11. In order to speed up the search for hyperparameters for the KRR models, we use a fixed regularization hyperparameter value of $\lambda = 2.22 * 10^{-16}$, while only optimizing the kernel width σ. While this may not yield optimal results, in our experience it does not affect the performance significantly, while greatly speeding up the computation time required for cross-validation. Supplementary Tables 12, 13, 14, and 15 show the resulting optimized values for the kernel width σ across the different models and molecules.

Supplementary Table 11: Hyperparameter values for the artificial Gaussians potential selected by cross-validation for every molecule.

Parameters/Molecule	Water	Ethanol	Benzene	Resorcinol
Grid spacing	0.33	0.19	0.20	0.20
Gaussian width γ	0.60	0.36	0.42	0.42

Supplementary Table 12: Kernel width parameters selected by cross-validation for the different KRR models for the water dataset.

	Water			
Parameter/Model	$n_{\mathrm{ML}}^{\mathrm{DFT}}$	$E_{\mathrm{ML}}^{\mathrm{DFT}}$	$E_{\mathrm{ML}}^{\mathrm{CC}}$	$E_{\Delta\text{-DFT}}^{\mathrm{CC}}$
Kernel width σ	7.98	20982	30738	49397629

Supplementary Table 13: Kernel width parameters selected by cross-validation for the different KRR models for the ethanol dataset.

	Ethanol			
Parameter/Model	$n_{\mathrm{ML}}^{\mathrm{DFT}}$	$E_{\mathrm{ML}}^{\mathrm{DFT}}$	$E_{\mathrm{ML}}^{\mathrm{CC}}$	$E_{\Delta\text{-DFT}}^{\mathrm{CC}}$
Kernel width σ	423.9	126976.19	126976.19	409446.22

Supplementary Table 14: Kernel width parameters selected by cross-validation for the different KRR models for the benzene dataset.

	Benzene			
Parameter/Model	$n_{\mathrm{ML}}^{\mathrm{DFT}}$	$E_{\mathrm{ML}}^{\mathrm{DFT}}$	$E_{\mathrm{ML}}^{\mathrm{CC}}$	$E_{\Delta\text{-DFT}}^{\mathrm{CC}}$
Kernel width σ	462.7	36308.7	81415.9	165160.4

Supplementary Table 15: Kernel width parameters selected by cross-validation for the different KRR models for the resorcinol dataset.

	Resorcinol			
Parameter/Model	$n_{\mathrm{ML}}^{\mathrm{DFT}}$	$E_{\mathrm{ML}}^{\mathrm{DFT}}$	$E_{\mathrm{ML}}^{\mathrm{CC}}$	$E_{\Delta\text{-DFT}}^{\mathrm{CC}}$
Kernel width σ	302.74	160793.66	345085.9	955296.68

3.4 Multiple time-step molecular dynamics in the ML framework

The generation of a molecular dynamics (MD) trajectory via numerical solution of the equations of motion $\dot{\mathbf{R}}_\alpha = \mathbf{P}_\alpha/M_\alpha$, $\dot{\mathbf{P}}_\alpha = \mathbf{F}_\alpha$, possibly also coupled to a thermostat, where $\alpha = 1, ..., N$ indexes the N atoms, M_α is the mass of the αth atom, $\mathbf{P}_\alpha$ is its momentum, $\mathbf{R}_\alpha$ is its position, and $\mathbf{F}_\alpha$ is the force on this atom. In DFT, if the true external potential is $v_{\rm ext}(\mathbf{r}, \mathbf{R})$, where $\mathbf{R}$ denotes the full set of atomic coordinates, then the force is given by

$$\begin{aligned} \mathbf{F}_\alpha &= -\int \mathrm{d}\mathbf{r}\; n(\mathbf{r})\nabla_\alpha v_{\rm ext}(\mathbf{r}, \mathbf{R}) + \mathbf{F}_{\alpha,\rm NN} \\ &\equiv \mathbf{F}_\alpha^{(\rm elec)} + \mathbf{F}_{\alpha,\rm NN} \end{aligned} \quad (12)$$

where $\mathbf{F}_{\alpha,\rm NN}$ is the force due to the nuclear-nuclear electronic repulsion. The first term refers to the force originating from the electron-nuclear interaction. In the machine-learning framework, the nesting of functional dependencies, leading to the progression $\mathbf{R} \to v \to n \to E$, such that the electronic force is given by

$$\mathbf{F}_\alpha^{(\rm elec)} = -\int \mathrm{d}\mathbf{r}\; \mathrm{d}\mathbf{r}'\; \frac{\delta E_{\rm ML}}{\delta n_{\rm ML}(\mathbf{r})} \frac{\delta n_{\rm ML}(\mathbf{r})}{\delta v(\mathbf{r}', \mathbf{R})} \nabla_\alpha v(\mathbf{r}', \mathbf{R}) \quad (13)$$

In a standard MD calculation, the numerical integration algorithm for generating P steps of MD using a time step Δt, is structured according to the pseudocode shown below:

$$\begin{aligned} &\text{for } i \text{ in range}(P) \\ &\quad \text{for } \alpha \text{ in range}(N) \\ &\qquad \mathbf{P}_\alpha \leftarrow \mathbf{P}_\alpha + \Delta t * \mathbf{F}_\alpha/2 \\ &\qquad \mathbf{R}_\alpha \leftarrow \mathbf{R}_\alpha + \Delta t * \mathbf{P}_\alpha/M_\alpha \\ &\qquad \text{Update Forces} \\ &\qquad \mathbf{P}_\alpha \leftarrow \mathbf{P}_\alpha + \Delta t * \mathbf{F}_\alpha/2 \end{aligned} \quad (14)$$

The obvious bottleneck in an MD calculation, which often restricts the value of P, *i.e.,* the time scale that can be accessed, is the computational overhead associated with the force calculation, as each step requires a full calculation of $\mathbf{F}_\alpha$. The computational time required to generate an MD trajectory can be reduced if the force can be subdivided into a component that has a low computational overhead and a correction that varies on a slower time scale and carries most of the computational overhead of the full force calculation. Denoting the former of these as a reference for $\mathbf{F}_\alpha^{(\rm ref)}$ and the correction as $\delta\mathbf{F}_\alpha$, the full force is then $\mathbf{F}_\alpha = \mathbf{F}_\alpha^{(\rm ref)} + \delta\mathbf{F}_\alpha$. With this force decomposition, a reversible, symplectic multiple time-step integration algorithm can be constructed [21] based on the assumption that the correction $\delta\mathbf{F}_\alpha$ only needs to be updated every m steps, where typically $m \sim 5$, which, if the computational overhead of the reference force calculation is negligible compared to the correction, will reduce the computational cost of the calculation by a factor of m. The algorithm, called the reversible reference system propagator algorithm (reversible RESPA), has the same structure as that shown in Supplementary Eq. (14) with the following provision: In each step, the default is to use $\mathbf{F}_\alpha^{(\rm ref)}$ in place of $\mathbf{F}_\alpha$ in each step, and the force update is only an update of the reference force; every m steps, however, one uses the force $\mathbf{F}_\alpha^{(\rm ref)} + m\delta\mathbf{F}_\alpha$, and the force update requires calculation of both the reference force and the correction.

In our Δ-machine learning approach, a natural force decomposition arises from the expression for the energy

$$\begin{aligned} E^{\rm CC}[n] &= E^{\rm DFT}[n] + \Delta E_{\rm ML}[n] \\ &= E^{\rm CC}_{\rm ML}[n] + \left(E^{\rm DFT}[n] + \Delta E_{\rm ML}[n] - E^{\rm CC}_{\rm ML}[n]\right) \\ &\equiv E^{\rm CC}_{\rm ML}[n] + \delta E_{\rm ML}[n] \end{aligned} \quad (15)$$

where the density $n(\mathbf{r})$ is either the explicit PBE density or the density from the Hohenberg-Kohn map. Note that, in the second line, we have added and subtracted the direct CCSD(T) ML model. We, thus, associated the force obtained from $E_{\mathrm{ML}}[n]$ with the reference force $\mathbf{F}_{\alpha}^{(\mathrm{ref})}$, the computational overhead of which is quite low. We then associate the correction $\delta\mathbf{F}_{\alpha}$ with the energy correction $\delta E_{\mathrm{ML}}[n]$. As this term requires a full DFT calculation, its computational overhead is significantly higher, and the overall reduction in computational time is very nearly equal to the value of m.

Supplementary references

[1] H.-J. Werner, P. J. Knowles, G. Knizia, F. R. Manby, M. Schütz, et al. Molpro, version 2015.1, a package of ab initio programs, 2015.

[2] Frank Neese. The ORCA program system. *Wiley Interdisciplinary Reviews: Computational Molecular Science*, 2(1):73–78, 2012.

[3] Thom H. Dunning. Gaussian basis sets for use in correlated molecular calculations. I. the atoms boron through neon and hydrogen. *The Journal of Chemical Physics*, 90(2):1007–1023, 1989.

[4] Stefan Chmiela, Alexandre Tkatchenko, Huziel E. Sauceda, Igor Poltavsky, Kristof T. Schütt, and Klaus-Robert Müller. Machine learning of accurate energy-conserving molecular force fields. *Science Advances*, 3(5):e1603015, 2017.

[5] Stefan Chmiela, Huziel E. Sauceda, Klaus-Robert Müller, and Alexandre Tkatchenko. Towards exact molecular dynamics simulations with machine-learned force fields. *Nature Communications*, 9(1):3887, 2018.

[6] M.J. Frisch, G.W. Trucks, H.B. Schlegel, G.E. Scuseria, M.A. Robb, et al. Gaussian 09, 2009.

[7] Kristof T. Schütt, Huziel E. Sauceda, P.-J. Kindermans, A. Tkatchenko, and K.-R. Müller. SchNet–a deep learning architecture for molecules and materials. *The Journal of Chemical Physics*, 148(24):241722, 2018.

[8] Mikio L Braun, Joachim M Buhmann, and Klaus-Robert Müller. On relevant dimensions in kernel feature spaces. *Journal of Machine Learning Research*, 9(Aug):1875–1908, 2008.

[9] Bernhard Schölkopf, Alexander Smola, and Klaus-Robert Müller. Nonlinear component analysis as a kernel eigenvalue problem. *Neural computation*, 10(5):1299–1319, 1998.

[10] P. Hohenberg and W. Kohn. Inhomogeneous electron gas. *Phys. Rev.*, 136(3B):B864–B871, Nov 1964.

[11] W. Kohn and L. J. Sham. Self-consistent equations including exchange and correlation effects. *Phys. Rev.*, 140(4A):A1133–A1138, Nov 1965.

[12] R. G. Parr and W. Yang. *Density Functional Theory of Atoms and Molecules*. Oxford University Press, 1989.

[13] Adam Wasserman, Jonathan Nafziger, Kaili Jiang, Min-Cheol Kim, Eunji Sim, and Kieron Burke. The importance of being inconsistent. *Annual Review of Physical Chemistry*, 68(1):555–581, 2017.

[14] Eunji Sim, Suhwan Song, and Kieron Burke. Quantifying density errors in DFT. *J. Phys. Chem. Lett.*, 9:6385–6392, October 2018.

[15] Michael G. Medvedev, Ivan S. Bushmarinov, Jianwei Sun, John P. Perdew, and Konstantin A. Lyssenko. Density functional theory is straying from the path toward the exact functional. *Science*, 355(6320):49–52, 2017.

[16] M. Levy and A. Görling. Correlation energy density-functional formulas from correlating first-order density matrices. *Phys. Rev. A*, 52:R1808, 1995.

[17] Li Li, John C. Snyder, Isabelle M. Pelaschier, Jessica Huang, Uma-Naresh Niranjan, Paul Duncan, Matthias Rupp, Klaus-Robert Müller, and Kieron Burke. Understanding machine-learned density functionals. *International Journal of Quantum Chemistry*, 116(11):819–833, 2016.

[18] Justin S Smith, Benjamin T Nebgen, Roman Zubatyuk, Nicholas Lubbers, Christian Devereux, Kipton Barros, Sergei Tretiak, Olexandr Isayev, and Adrian E Roitberg. Approaching coupled cluster accuracy with a general-purpose neural network potential through transfer learning. *Nature communications*, 10(1):1–8, 2019.

[19] Trevor Hastie, Robert Tibshirani, and J.H. Friedman. The elements of statistical learning: Data mining, inference, and prediction, 2009.

[20] Katja Hansen, Grégoire Montavon, Franziska Biegler, Siamac Fazli, Matthias Rupp, Matthias Scheffler, O. Anatole von Lilienfeld, Alexandre Tkatchenko, and Klaus-Robert Müller. Assessment and validation of machine learning methods for predicting molecular atomization energies. *J. Chem. Theory Comput.*, 9(8):3404–3419, 2013.

[21] Mark E. Tuckerman, Bruce J. Berne, and Glenn J. Martyna. Reversible multiple time scale molecular dynamics. *The Journal of Chemical Physics*, 97(3):1990–2001, 1992.
